# Self-assembly, optical and electrical properties of perylene diimide dyes bearing unsymmetrical substituents at bay position

**DOI:** 10.1038/s41598-018-26502-5

**Published:** 2018-05-29

**Authors:** Fengxia Zhang, Yongshan Ma, Yanhui Chi, Haohai Yu, Yanan Li, Tianyi Jiang, Xiaofeng Wei, Jingmin Shi

**Affiliations:** 1 College of Chemistry, Chemical Engineering and Materials Science, Collaborative Innovation Center of Functionalized Probes for Chemical Imaging in Universities of Shandong, Key Laboratory of Molecular and Nano Probes, Ministry of Education Shandong Provincial Key Laboratory of Clean Production of Fine Chemistry, Shandong Normal University, jinan, 250014 P.R. China; 2 School of Municipal and Environmental Engineering, Shandong Jianzhu University, Jinan, 250101 P.R. China; 3 State Key Laboratory of Crystal Materials Shandong University Jinan, Jinan, 250100 P.R. China; 4 Shandong Provincial Key Laboratory of Metrology and Measurement, Shandong Institute of Metrology, Shandong Social Justice Institute of Metrology, Jinan, 250014 P.R. China; 5 Co-Innovation Center of Green Building, Jinan, 250101 P.R. China

## Abstract

Perylene diimides (PDIs) are one class of the most explored organic fluorescent materials due to their high luminescence efficiency, optoelectronic properties, and ready to form well-tailored supramolecular structures. However, heavy aggregation caused quenching (ACQ) effect in solid state has greatly limited their potential applications. We have easily solved this problem by chemical modification of the PDI core with only phenoxy moietie at one of the bay position. In this paper, we report two perylene bisimides with small rigid substituents, 1- phenol -N, N’-dicyclohexyl perylene-3,4,9,10-tetracarboxylic diimide (PDI **1**) and 1- p-chlorophenol-N, N’-dicyclohexyl perylene-3,4,9,10-tetracarboxylic diimide (PDI **2**) possess both well defined organic nanostructures and high fluorescence quantum yield in the solid state. In contrast, 1-propanol-N, N’-dicyclohexyl perylene-3,4,9,10-tetracarboxylic diimide (PDI **3**) bearing a straight chain only shown weak orange fluorescence. In addition, morphological inspection demonstrated that PDI **3** molecules easily form well-organized microstructures despite the linkage of the PDI core with a straight chain. The present strategy could provide a generic route towards novel and advanced fluorescent materials and these materials may find various applications in high-tech fields.

## Introduction

Perylene diimides (PDIs) are one kind of the most investigated organic dyes because of their excellent chemical, thermal, and optical stabilities, as well as their high luminescence efficiency and novel optoelectronic properties^1–5^. They behave as n-type organic semiconductor materials and have been used in fabrication of solar cells, photovoltaic devices, dye lasers, and so on^6,7^ .Because of strong π-π interaction between perylene cores, PDI derivatives are prone to assemble into ordered 1D nanostructure such as rods, wires, and belts^8,9^ .In dilute solutions, PDIs demonstrate near-unity fluorescence quantum yields, but in solid state, PDI molecules are prone to form aggregates, which lead to fluorescence quenching because of the effective intermolecular π-π actions and/or attractive dipole–dipole interactions. This effect is called aggregation-caused quenching (ACQ)^10^. It is of very important to make PDI-based dyes efficiently emit in the solid state^11–16^. A sensible strategy is to modify the perylene chromophore with bulky substituents to hinder the intermolecular electronic coupling and to restrict the intermolecular π-π stacking between perylene cores. The resulting PDIs can exhibit desirable quantum efficiency of light emission^17–19^.

Self assembly of PDIs to form ordered supramolecular architectures have received increased attention, especially with the development of electronic devices, where they will find the application of new electronic materials^20,21^ .The molecular architecture and electrical properties are closely related in semiconducting organic electronic materials^22^. Side chain of PDIs play a vital role in controlling the molecular stacking and it affects the color and other physico-chemical properties of the PDI aggregates. Obviously, the spectral change in the solid state is the exhibition of an aggregation effect of the molecules. This sensitive side-chain modulation provides a way to control and optimize the molecular packing conformation so that the self-assembly 1D growth can be realized. Incorporating substituents to the imide nitrogen atoms in PDI skeleton can increase the solubility of PDI derivatives in organic solvents and help the packing of PDI molecules as nanostructures^23^. However, substitution at the imide position can not affect the properties and electronic structures of PDIs, because the nodes in both lowest unoccupied molecular orbital (LUMO) and highest occupied molecular orbital (HOMO) levels limit the electronic interactions between PDI and corresponding substituents^24^. Many literatures regarding modification of PDI derivatives at imide positions to generate self-assembled nanostructures have been reported^25,26^. However, few literature reported the self-assembly of bay area substituted PDI derivatives although substituents at bay area can dramatically change the electronic structures and photochemical properties of PDIs^27^. It is because that this kind of PDIs was difficult to form well-defined nanostructures due to the distorting π-π stacking^28–30^.

**Figure 1:**
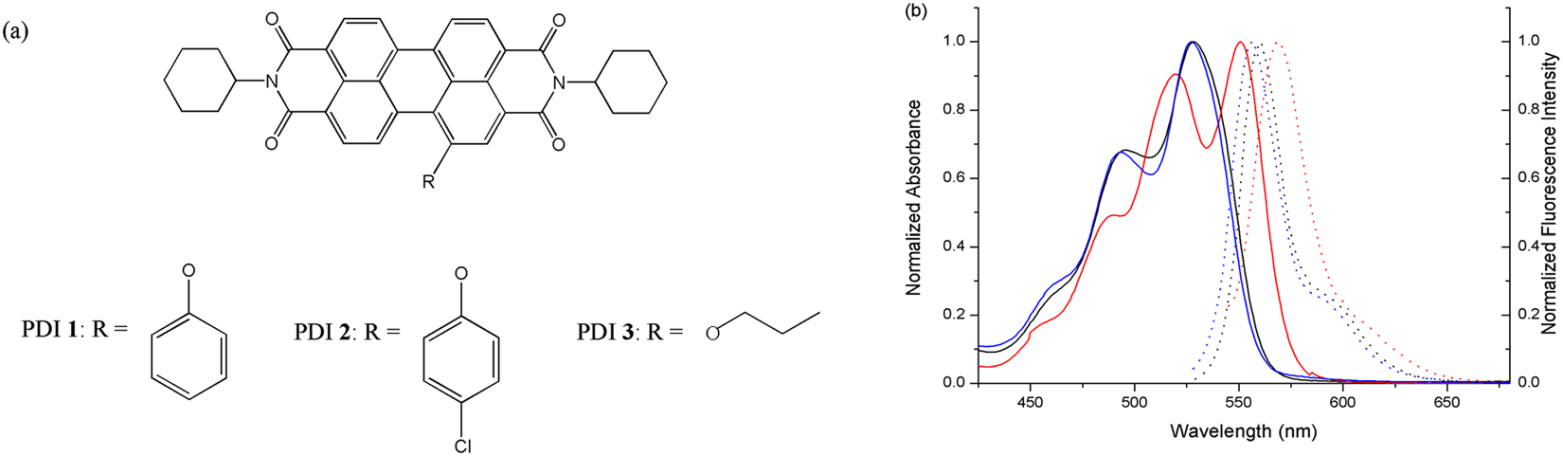
Molecular design and optical properties of PDIs **1**, **2** and **3**. (**a**) Chemical structures of PDIs **1** and **2**. (**b**) Absorption spectra (solid lines) and corresponding fluorescence spectra (dot lines) of PDI **1** (black), PDI **2** (blue) and PDI **3** (red) in DCM.

Herein, we report the design and synthesis of two PDIs bearing unsymmetrical phenoxy substituents: 1-phenol -N, N′-dicyclohexyl perylene-3,4,9,10-tetracarboxylic diimide (PDI **1**), 1-p-chlorophenol-N, N′-dicyclohexyl perylene-3,4,9,10-tetracarboxylic diimide (PDI **2**) and PDIs with n-propoxy substituent at bay position: 1-propanol-N, N′-dicyclohexyl perylene-3,4,9,10-tetracarboxylic diimide (PDI **3)** (Fig. 1). Because of the weak intramolecular hydrogen bond, PDIs **1** and **2** have an approximate planar configuration. This may increase conjugation between the PDI cores with the substituent. Aggregation of the molecules, which is dominated by the PDI rings and the benzene segment, results in a highly ordered PDI packing and benzene packing in the solid state. The attachment of phenol or p-chlorophenolat at bay area may partly distort the π-π stacking between the PDI units, and PDIs **1** and **2** can easily possess both well defined organic nanostructures and high fluorescence quantum yield. Through the analysis of their crystal structures, the effect of heteroatom substitute phenoxy segment on the assembly of perylene diimide was investigated. In contrast, the aggregation of PDI **3** was influenced by the straight chain at bay position. The propanol chain of **3** results in a disordered packing, cuts down the packing order of PDI units in the molecular packing, and the intermolecular π-π actions are weaker. Because of the effect of ACQ, the fluorescence is very weak.

## Results and Discussion

### Molecular design and optical properties

The molecular structures of PDIs **1**, **2**, and **3** are shown in Fig. 1a. The usual strategy for introducing a group into the PDI core is using substitution reactions with mono-halogenated PDIs as starting materials. The bromination or chlorination of PDIs could be easily achieved but the reactions would result in a mixture of various PDIs halogenated at different levels. It was very difficult to separate the mixture owing to the presence of regioisomers and their bad solubilities. The main products of replacement of the halogen atoms are di- (even tetra-) substituted symmetrical PDIs^31,32^. The low activities of halogenated PDIs make some reactions difficult to be carried out. Owing to the strong electron-withdrawing effect of nitro group, the mono-nitrated PDI can be easily achieved under ambient temperature with a high yield of 90%. The synthesis started from imidization of perylene bisanhydride by reaction with cyclohexylamine. The compound **5** was achieved by a reaction of compound **4** with Ce(NH_4_)_2_(NO_3_)_6_ and HNO_3_ at 25 °C in dichloromethane with a high yield. The substitutions of nitro group of **5** with phenol, p-chlorophenol group or n- propanol were performed to obtain the corresponding substituted products **1**, **2**, or **3**. The substituents at bay position of PDIs **1** and **2** are aromatic nucleus, while that of PDI **3** is linear chain. The structures of compounds **1**, **2**, and **3** have been fully characterized through nuclear magnetic resonance (^1^H NMR for compound **1** and ^1^H and ^13^C NMR for compound **2** and **3**), Fourier transform infrared spectroscopy (FT-IR) and mass spectroscopy (MS) (see the Supporting information). Because of the poor solubility of compound **1**, we cannot observe its ^13^CNMR spectrum.

These bay substituents also influence the optical properties of PDIs. The respective absorption and fluorescence data are listed in Table 1. PDIs **1** and **2** have a similar length of conjugation. Therefore, they exhibit similar absorption spectra (Fig. 1b). The spectra of **1** show two absorption bands (528 nm, 496 nm) and a broad shoulder peak around 460 nm, which are in accord with the characteristics of the 0–0, 0–1, and 0–2 transition energy^29^. Also, these spectra conform to the absorption peaks of **2** appeared at 527 nm, 493 nm, and a shoulder around 461 nm. The longest maximum for **3** (552 nm) is red-shifted relative to PDIs **1** and **2**. Such a change can be caused either by the electronic coupling between the electron-deficient PDI core and the electron richer substituent, or substituent induced distortion of PDI core^27^.

**Table 1:** Absorption and fluorescence data of PDIs 1, 2 and 3 were measured ^a^in solutions (10 μM in dichloromethane) and ^b^in the solid states.

compounds	^a^Abs.λ (nm)	^a^PL λem. (nm)	^a^Φ (%)	^a^⟨τ⟩ (ns)	^b^PL λem. (nm)	^b^Φ (%)
PDI **1**	528, 496, 460	560, 595	57.2	5.51	691	19.2
PDI **2**	527, 493, 461	557, 591	52.6	5.30	690	14.5
PDI **3**	552, 521, 490	568, 610	43.5	5.43	700	6.2

The fluorescence quantum yield in solutions (Φ) was evaluated in dichloromethane solution by using fluorescein as standard (Φ_F_ = 0.85, 0.1 M NaOH).

The fluorescence spectra of PDIs **1**, **2**, and **3** in DCM depict the same structure with approximate mirror images of the absorption spectrum, and the emission peaks are appeared at 560 nm, 557 nm, and 568 nm for **1**, **2**, and **3**, with corresponding Stokes shifts of 32 nm, 30 nm, and 16 nm, respectively. This indicated that the Stokes shift decreased along with the introduction of chlorinatomin or phenoxy to the electron rich ether substituent. The fluorescence emission spectra of PDIs **1**, **2**, and **3** in solid state are shown in Fig. S2. The fluorescence emission peak of the solid-state structure was observed at 691 nm, 690 nm, and 700 nm for **1**, **2**, and **3**, respectively. The red shifts (163 nm, 163 nm, and 132 nm for **1**, **2**, and **3**, respectively) of the emission peak positions can be ascribed to the excimer formation of the PDIs motif in solid state.

The emission quantum yields (Φ) in dichloromethane solution^33^ and solid state are 57.2%, 19.2% for **1**, 52.6%, 14.5% for **2**, and 43.5%, 6.2% for **3**, respectively. Thus, PDIs **1**, **2**, and **3** exhibit a certain degree of ACQ. To interpret the photophysical properties in a more intuitive manner, the fluorescence lifetimes of PDIs **1**, **2**, and **3** in dichloromethane solvent were measured and shown in Fig. S4. The results of lifetime measurements of PDIs **1**, **2**, and **3** are listed in Table 1. The PDIs **1** and **3** have single lifetime and decays were mono-exponential and the average fluorescence lifetimes are 5.51 ns and 5.43 ns, respectively. While PDI **2** gives a fluorescence lifetime with double exponential decay feature (τ_1 = _5.30 ns, 81%; τ_2_ = 500.0 ns, 19%).

**Figure 2:**
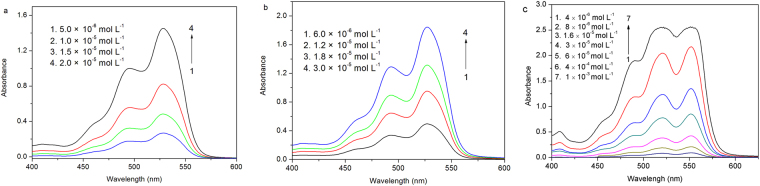
Absorption spectra of **1** (**a**), **2** (**b**) and **3** (**c**) in dichloromethane at various concentrations.

The absorption spectra of **1**, **2**, and **3** in dichloromethane at various concentrations are shown in Fig. 2. As free monomers, the normal progression of Franck–Condon factor is *A*^0–0^ > *A*^0–1^ > *A*^0–2^. However, when the monomer begin to aggregate, the 0–1 transition increases^29^. 0–1 transition absorptions of **1** (from 5.0 × 10^−6^ to 2.0 × 10^−5^ M) and **2** (from 6.0 × 10^−6^ to 3.0 × 10^−5^ M) increased obviously (Fig. S3a,b), while that of **3** (from 10^−6^ to 6.0 × 10^−5^ M) did not show any increase in dichloromethane (Fig. S3c). These results suggest that the aggregation occurred for **1** and **2** but didn’t occur for **3** in low concentration. This trend indicates that the flexibility n-propoxy substituent can effectively improve the solubility of the rigid perylene dye and inhibit the aggregation of perylene cores by shielding the π faces.

**Figure 3:**
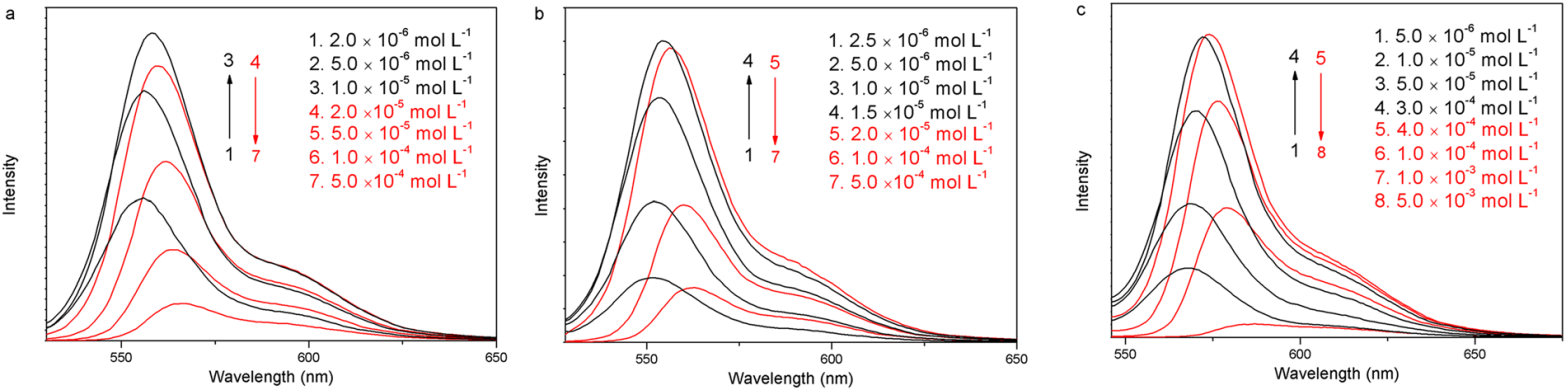
Fluorescence spectra of **1** (**a**), **2** (**b**) and **3** (**c**) in dichloromethane at various concentrations.

The emission spectra of **1**, **2**, and **3** in dichloromethane at all the concentrations show that the peaks are mirror images of the absorption spectra (Fig. 3). Unlike the UV results, the fluorescence intensity increased linearly at concentration ranging from 2.0 × 10^−6^ M to 1.0 × 10^−5^ M for **1**, 2.5 × 10^−6^ M to 1.5 × 10^−5^ M for **2**, and 5.0 × 10^−6^ M to 3.0 × 10^−4^ M for **3** before they continuously decreases as the concentration increases from 2.0 × 10^−5^ M to 5.0 × 10^−4^ M for **1**, 2.0 × 10^−5^ M to 5.0 × 10^−4^ M for **2**, and 4.0 × 10^−4^ M to 5.0 × 10^−3^ M for **3**. Meanwhile, the maximum absorption bands shift from 555 nm to 566 nm for **1**, 551 nm to 563 nm for **2**, and 567 nm to 585 nm for **3**. These results indicate that self-quenching and aggregation occured in the perylene cores at high concentrations.

**Figure 4:**
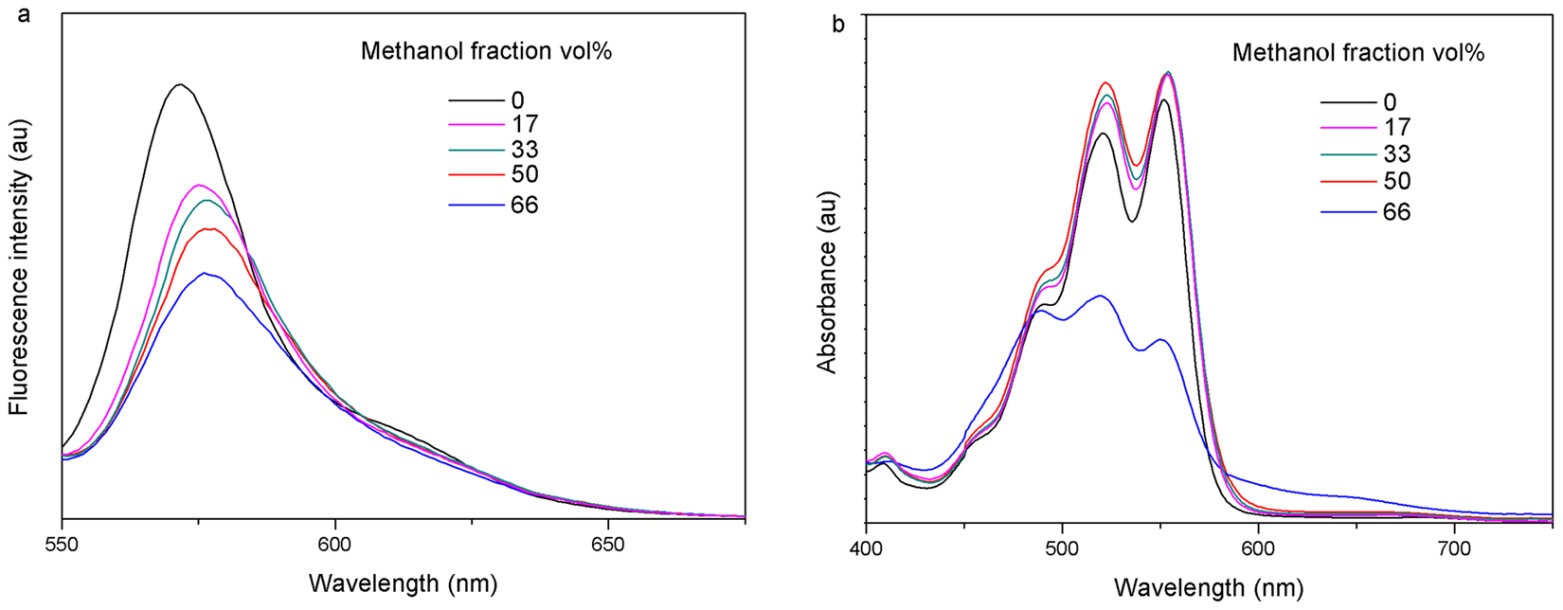
Fluorescence spectra (**a**) and absorption spectra (**b**) of 3 in methanol/dichloroform mixtures with different methanol fractions (ƒ_w_, by volume%).

The self-assembly of molecule **3** was further investigated in methanol/dichloroform mixtures with different methanol fractions (ƒ_w_). The fluorescence spectrum of PDI **3** in dichloroform solution (10^−5^ M) shows a peak at 568 nm (Fig. 4a). With the increase of ƒ_w_, the emission intensity decreased. When ƒ_w_ < 17%, the change in emission intensity is decreased to 77%, and the emission peak moved to 575 nm. When 17% < ƒ_w_ < 33%, the change in emission intensity was rather small (only about 4%), and the emission peak shifted to 576 nm. The fluorescence intensity of PDI **3** decreased gradually with the increase of ƒ_w_, and the maximum band of PDI **3** shifted to 577 nm. The absorption bands at 552 nm and 523 nm increased gradually with the increase of ƒ_w_ from 0% to 50% (Fig. 4b). The absorption bands decreased drastically and the relative intensity of 0–0 and 0–1 bands reverses as ƒ_w_ = 66%, indicating the aggregation occur. Self-assemblies with hypsochromic shifts of absorption bands are commonly termed as H-aggregates^34^.

### Self-assembled structures

The fabrication was done through a slow solvent-exchange process, in which the self-assembly of the molecules occurred at the interface between a “good” solvent (dichloroform) and a “poor” solvent (methanol). Typically, methanol (3.0 mL) was added dropwise into a concentrated dichloromethane solution of PDIs (6.0 mL, about 1.0 mg/mL) in a glass vial. Red aggregates were formed at the interface within minutes. Such a self-assembly approach takes the advantage of strong intermolecular π-π interaction, which is enhanced in a solvent where the solvophobic interaction of the molecule is maximized. Similar methods have previously been used for self-assembling of 1D nanostructure of planar aromatic molecules^35^.

**Figure 5:**
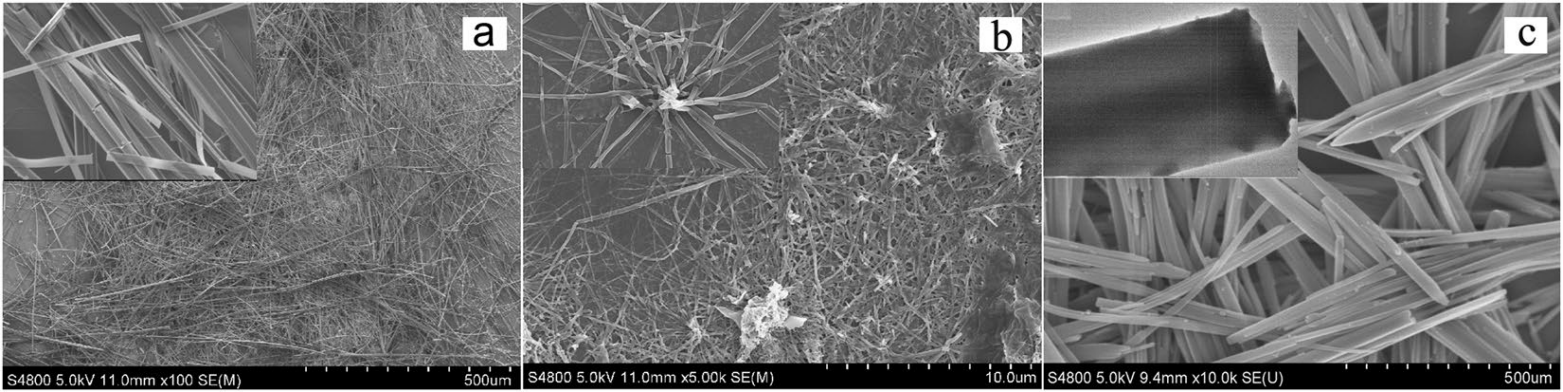
Self-assembled structures of PDI **1–3** deposited on a glass slide. (**a**) PDI **1** nanofibers. (**b**) PDI **2** nanofibers. (**c**) PDI **3** nanorods.

**Figure 6:**
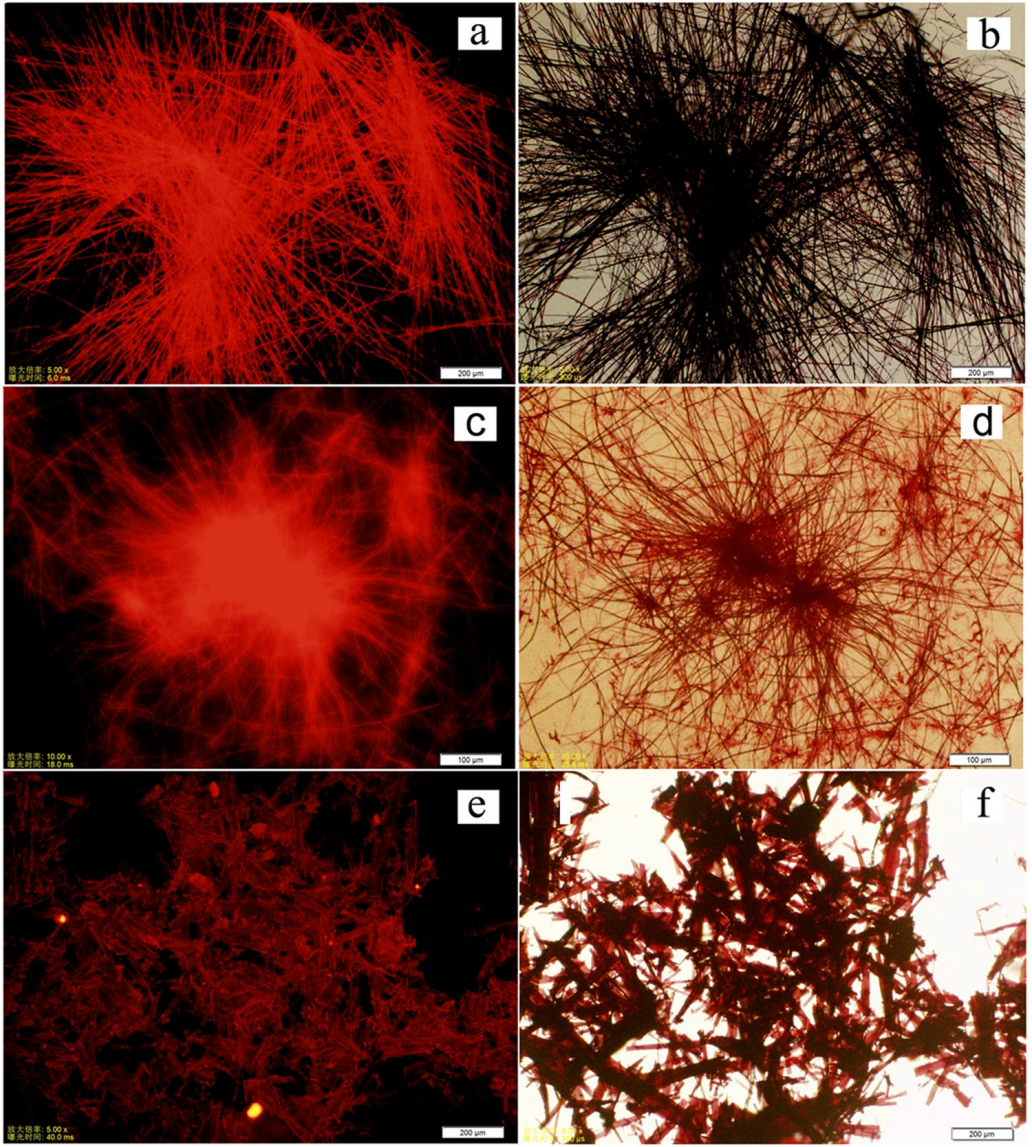
Fluorescence microscopic images of PDI **1–3** (**a**) PDI **1** nanobelt (**c**) PDI **2** nanofiber (**e**).

Figure 5a shows the SEM image of PDI **1** nanostructure. Long flexible microfiber with uniform morphology in terms of both width and thickness can be seen. The average width is 3 μm, and the length is in the range 300 μm to 500 μm. In contrast to PDI **1**, the SEM image of the PDI **2** shows that the molecules were assembled as 1D nanofibers. The average width is 100 nm, and the length is in the range within a few tens of micrometers (Fig. 5b). Figure 5c shows the SEM image of PDI **3** microrods with uniform morphology in terms of both width and thickness. The average width is 30 μm, and the length is in the range of a few hundreds of micrometers. The inset demonstrates that microstructures have regular edges and smooth surfaces, indicating good organized 1D microstructure have formed. The morphology was also supported by optical and fluorescence microscopic images (Fig. 6). As shown by the confocal fluorescence images, the phenoxy substituents of PDIs **1** and **2** made the microfibers and nanofibers emit intense red fluorescence upon excitation with green light (Fig. 6a,c). The bright red emission could be seen clearly in each fiber. In contrast, the confocal imaging measurement for PDI **3** only showed weak orange fluorescence because of the severe aggregation-caused quenching effect (Fig. 6e).

### Electronic structures

**Figure 7:**
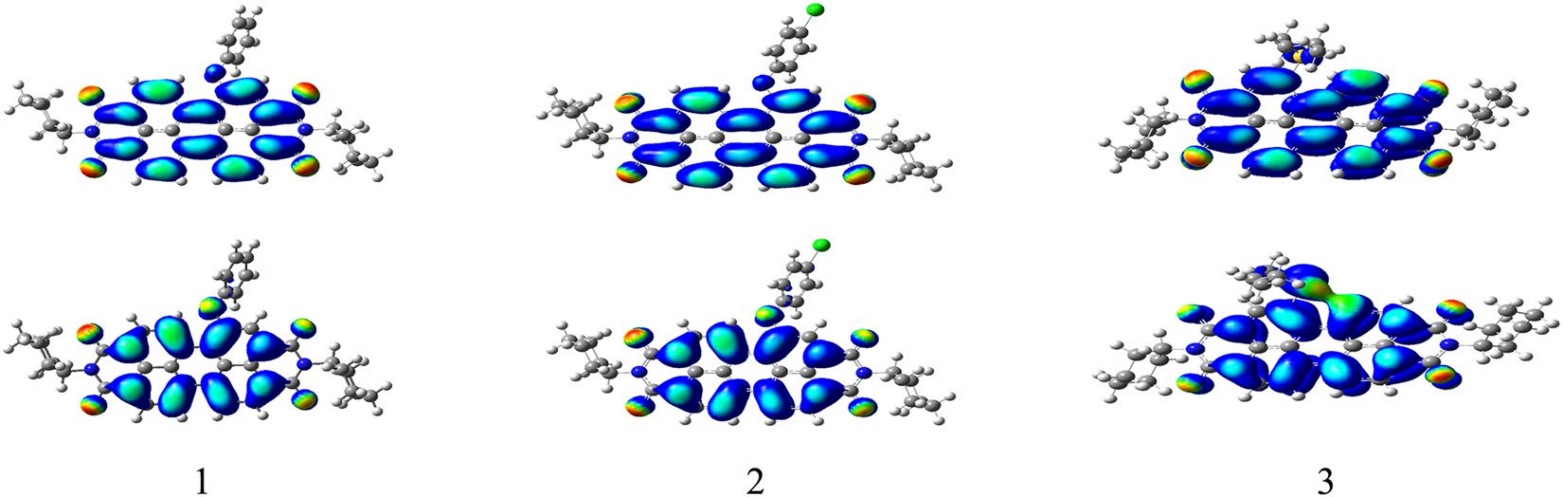
Computed frontier orbitals of PDIs **1**, **2** and **3**. The upper graphs are the LUMOs and the lower ones are the HOMOs.

To gain insight into the geometrical and electronic structures of the PDIs **1**, **2**, and **3**, chemical calculations were carried out with density functional theory (DFT) at the B3LYP/6-31 G^*^ level using the Gaussian03 package^36–38^. Figure 7 shows the LUMOs and the HOMOs of PDIs **1**–**3**. The calculation results reveal that the HOMO and LUMO orbitals of both **1** and **2** are localized predominately on the perylene ring system. The phenol and p-chlorophenol moieties contributed little to the molecular orbitals of **1** and **2**. It is notable that the HOMO orbital of **3** is delocalized on the perylene core and oxygen heteroatom site, while the LUMO orbital is centered on the perylene ring system. The energy gap between HOMO and LUMO is decreased for **3**. This is consistent with the red-shift of the electronic spectra for **3** compared with that of PDIs **1** and **2**.

**Figure 8:**
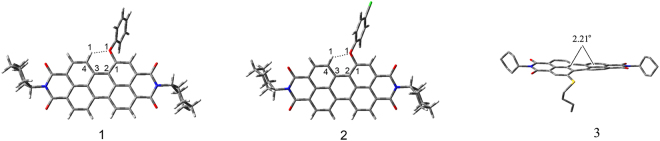
Optimized molecular structures of PDIs **1**, **2** and **3**.

According to the calculations, the distances between H_1_…O_1_ are 1.98 Å and 1.99 Å for **1** and **2**, respectively, and the bond angle of C_4_-H_1_…O_1_ are 124.7° and 124.5° for **1** and **2**, respectively (Fig. 8). These distances and angles accord with the range of weak hydrogen bond^27^. Furthermore, the C1-C2-C3-C4 dihedral angles are 5.84° and 6.47° for **1** and **2**, respectively. Because of the weak hydrogen bond between H_1_ and O_1_, PDIs **1** and **2** have an approximate planar configuration. This may increase conjugation between the PDI core with the substituent, and explain the observed ultralong 1D nanostructure for **1** and **2**. Approximate dihedral angles between the two naphthalene subunits attached to the central benzene ring, which was 0.46° for PDI **1** and 0.25^◦^ for **2**, both smaller than that of PDI **3** (2.21^◦^). The perylene skeleton has planar conformations, while the introduction of n-propoxy substituent into the perylene skeleton broke down the original conformation of perylene. The relatively high twisted dihedral angles of PDI **3** enhance its solubility, and cause significant effect on its optical properties and process ability in the solid state.

**Figure 9:**
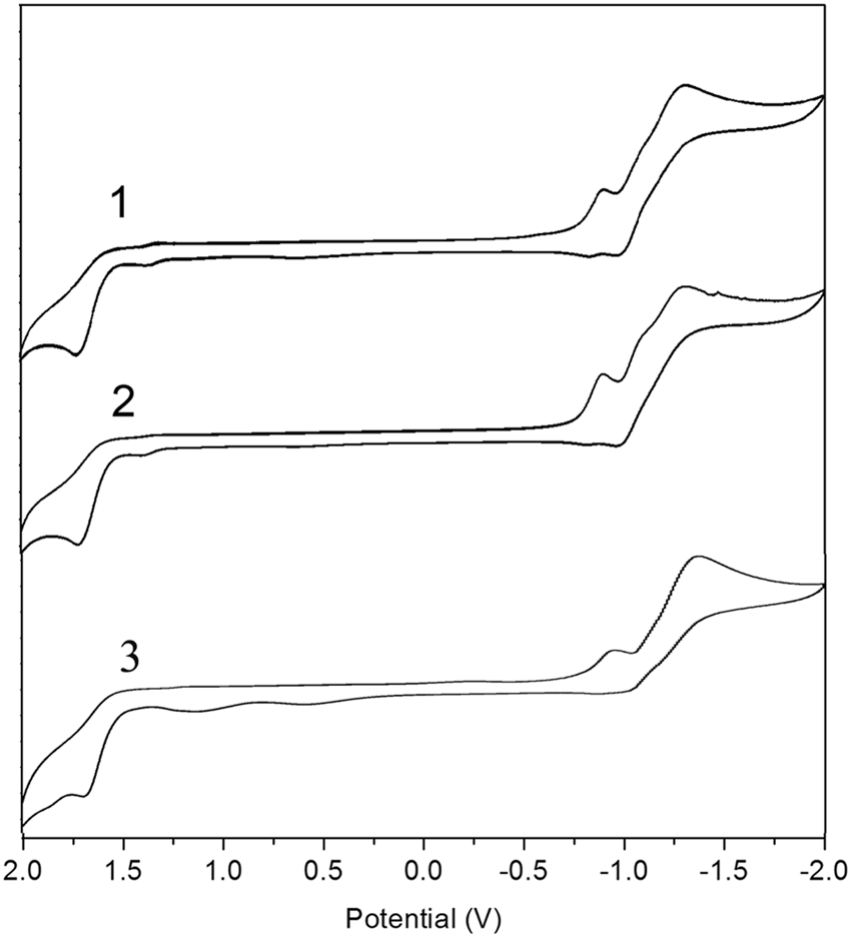
The cyclic voltammogram of PDIs 1–3 in CH_2_Cl_2_ (under N_2_ atmosphere, scanning rate 20 mV/s) using Bu_4_NPF_6_ as electrolyte, glassy carbon electrode as work electrode, platinum as the counter electrode, and Ag/AgNO_3_ as reference electrode.

Since the electronic structure of PDIs **1**–**3** is calculated to be dominated by the PDI core, it is expected that the n-type semiconductor behaviour will remain unchanged by the attachment of unsymmetrical substituents at bay position. This has been confirmed by experiment results of cyclicvoltammetry measurements (Fig. 9). In DCM solution, these chromophores show similar electrochemical behavior. As expected, they exhibit two quasi-reversible reduction waves and one quasi-reversible oxidation wave. These two reduction processes originate from successive reduction of the perylene core that give a radical anion in the first step, and a dianion in the second step^35^. The HOMO and the LUMO energy levels of **1**, **2** and **3** estimated from cyclic voltammetry were estimated to be −6.71/−4.10 eV, −6.72/−4.09 eV and −6.49/−3.96 eV respectively. These results indicate that PDI **1**–**3** have the same electrochemical behaviors as PDI and can be assigned to n-type organic semiconductors.

### X-ray diffraction study and molecular packing

**Figure 10:**
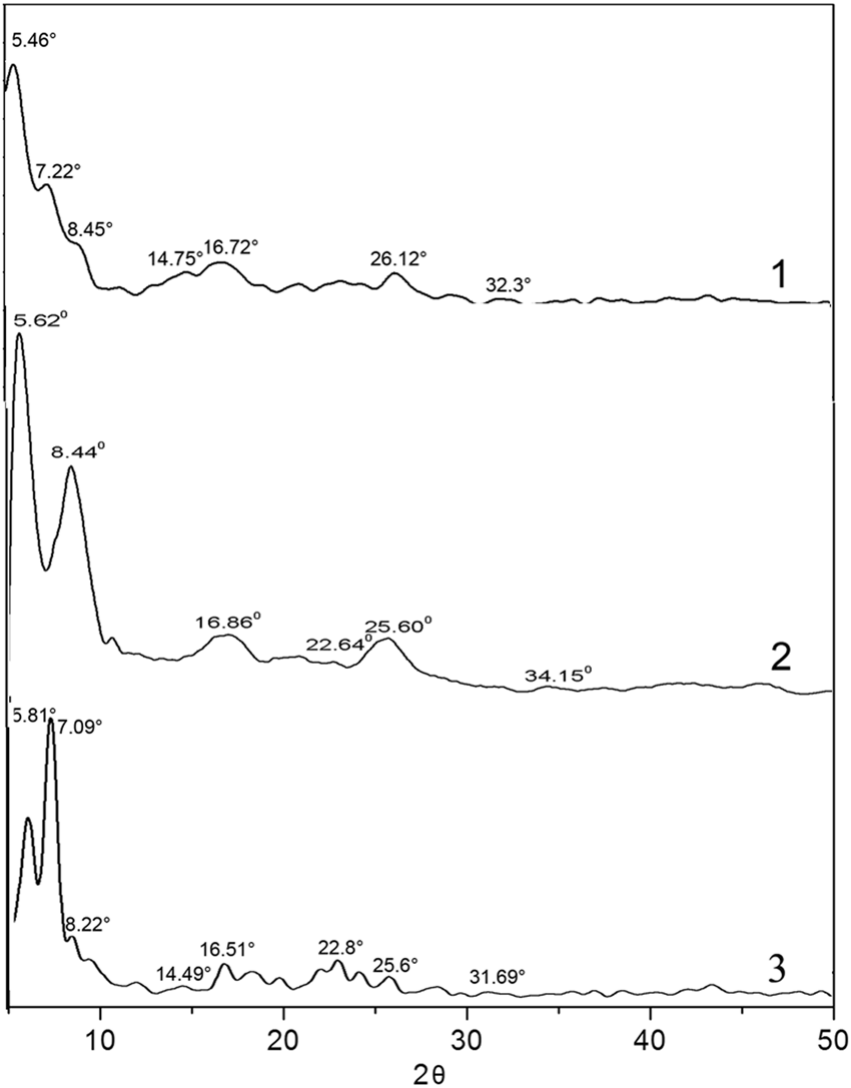
X-ray diffraction analysis of PDIs **1**, **2** and **3**.

To obtain information about the internal structure of the aggregates precipitated from solution. X-ray diffraction (XRD) experiments are performed on self-assembled PDIs **1**–**3** (Fig. 10). The diffraction patterns are complicated and cannot be assigned completely. In the XRD pattern of **1**, the peak at 26.12° corresponding to a spacing of 3.3 Å can be attributed to the π-π stacking of the adjacent PDIs because the distance of π-π stacking between the perylene cores is about 3.5 Å^39,40^ .In the low angle range, the XRD pattern of **1** shows a clear diffraction peak at 7.22° (1.22 nm), which correspond to the diffraction from the (010) plane^41^ .Compared with **1**, the peak shifts to 8.44° (10.46 Å) for **2**. The diffraction peaks at about 2θ = 5.46° (for **1**) and 5.62° (for **2**), which correspond to a space of about 1.61 nm and 1.56 nm, respectively, can be assigned to the length of the PDI units. The intensity of this diffraction peak decreased obviously for **3**. This result suggests that the n-propoxy substituent cut down the packing order of PDI units in the molecular packing. In addition, the XRD data of **3** displays a greater number of diffraction peaks between 15° and 30°. The peak at approximately 22.8° (3.8 Å) can be ascribed to the dislocation of π-π stacking of perylene. The intensity of diffraction peak at 25.6° (3.4 Å) (which can be attributed to the π-π stacking distance of PDI rings between the adjacent molecules) decreases obviously. This result suggests that the π-π stacking interactions of **3** units are weak. Additionally, XRD measurement of **1** indicates a first diffraction peak at 8.45° (10.25 Å) with the second order diffraction peak at 16.72° (5.18 Å) and fourth order diffraction peak at 32.3° (2.68 Å). A group of similar diffraction peaks found in the same region are at 8.44° (10.46 Å), 16.86° (5.24 Å), 34.15° (2.22 Å) for **2** and 8.22° (10.54 Å), 16.51° (5.23 Å), 31.69° (2.73 Å) for **3**, respectively. The first diffraction peak of **1** is assigned as (001) and the remaining higher order peaks as (00l) diffractions of the α-form crystal, which have been reported by Miyata^42^. A diffraction peak at 14.75° (5.87 Å) is observed in the diffraction pattern of **1**, which is shifted to 14.49° (5.97 Å) in the diffraction of **3** and can be attributed to the α-form crystal also. The multiple orders of reflection show that the self-assembled structures are layered and well ordered microstructures.

**Figure 11:**
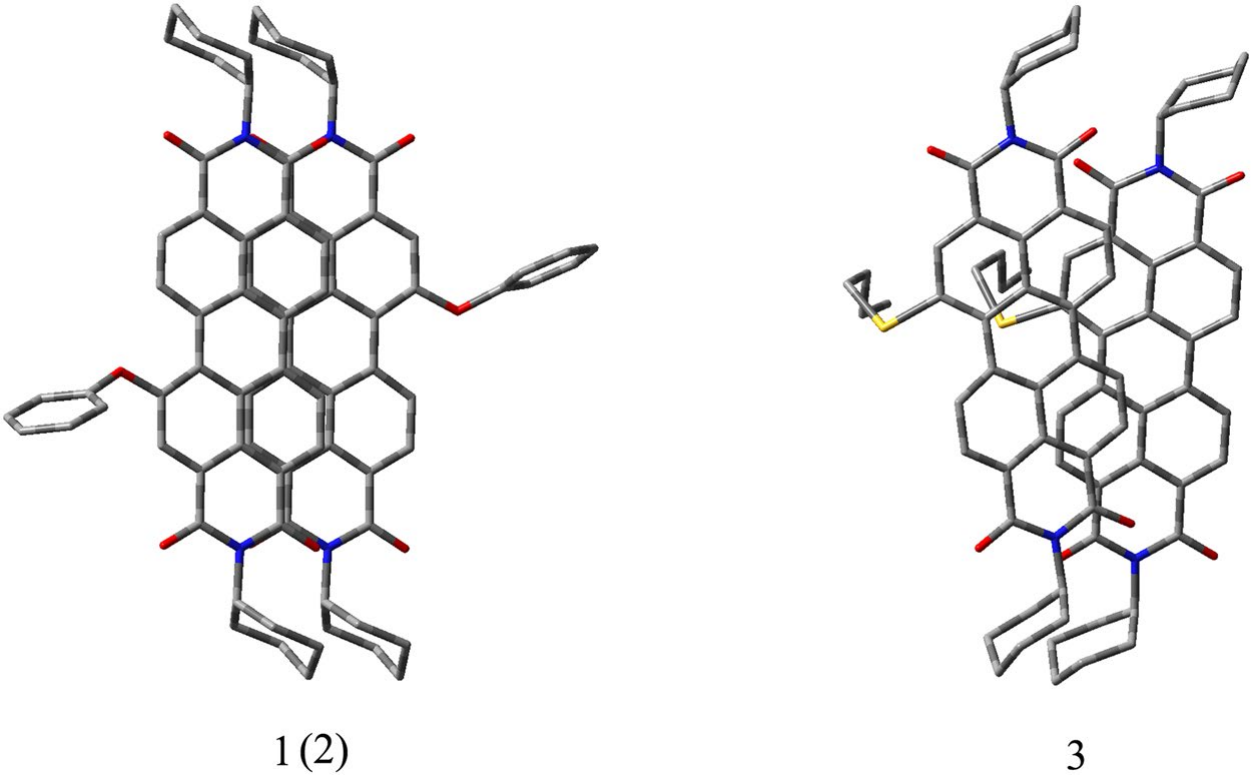
View of the single crystal structure of **1** and **3**. (The Becke’s three parameter gradient-corrected hybrid density function B3LYP method and the standard 6–31 G^*^ (d) basis set were used for the molecular arrangement in PDIs **1** and **3** solid based on two molecular models).

By comparing the XRD profiles, density functional theory (DFT), and the morphological analyses using SEM of aggregates **1**, **2**, and **3**, we can conclude that the aggregation of the molecules is dominated by the PDI rings with substituents at bay position (Fig. 11). Because of the weak intramolecular hydrogen bond, PDIs **1** and **2** have an approximate planar configuration. PDI **1** and **2** molecules remain the same face-to-face π–π packing as all known PDIs crystal structures. They adopt highly ordered PDI packing and benzene packing in the solid state. However, there is a transverse offset between adjacent PDI **1** and **2** molecules because of steric hindrance between the phenoxy/p-chlorophenoxy substituents at bay position. And about twelve carbon atoms in perylene ring are close to the adjacent perylene ring (a perylene ring is composed of 20 carbon atoms) and the overlapping area is limited, indicating extraessential intermolecular π–π actions and providing high luminescence efficiency. The interplanar distance between perylene moieties is found to be 3.3 Å for **1** and 3.4 Å for **2**. However, the aggregation of molecule **3** is influenced by the n-propoxy substituent at bay position, which results in a disordered ether chain packing and drives the PDI units to change their packing structure accordingly. From the molecular modeling studies it can be concluded that the oxygen atom of the n-propoxy substituent has actually help firming up the stacking of the molecules through non-covalent bonding. Such bonding might be electrostatic attraction between the electron-poor perylene core and the electron-rich atom O, or hydrogen bonds with H-O in CH_3_OH and dipole–dipole interactions. In either case, it also lead to fluorescence quenching of PDI **3**. The intermolecular π-π actions of **3** are weaker but the space between perylene chromophores is still very short (3.4 Å), which facilitate the hopping transportation of charge carrier from one perylene chromophore to a neighboring one. Thus, compound **3** could be a candidate material for acquiring well defined organic nanostructures with good charge-transporting.

## Conclusion

In this research, two PDIs bearing unsymmetrical phenoxy substituents and a PDI with n-propoxy substituent at bay position have been prepared successfully. The aggregations of the molecules are dominated by the PDI rings and the substituent segments. PDI **1** and PDI **2** adopt highly ordered PDI packing and benzene packing in the solid state. Due to the steric hindrance of phenoxy substituents substituent, the intermolecular π-π actions of PDI **1** and **2** are restricted, and PDI **1** and **2** nanostructures emit bright red fluorescence. The oxygen atom of the n-propoxy substituent in PDI **3** would help firming up the stacking of the molecules through electrostatic attraction and non-covalent interaction. The results reveal that introducing suitable substitute to a PDI derivative could introduce a special aggregation behavior for the PDI units during aggregation. Based on the advantages arising from the combination of classical PDI fluorogens and phenoxy moieties, we expect the present strategy could provide a generic route towards novel and advanced fluorescent materials and these materials may find various applications in high-tech fields.

## Experimental Section

### General procedure

All chemical reagents employed were of reagent grade and purchased from commercial source. Solvents were distilled through conventional procedures before used. Compound N, N′-dicyclohexyl-3, 4; 9, 10-tetracarboxylic acid diimide (**4**) and compound N, N′-dicyclohexyl-1-nitroperylene-3, 4; 9, 10-tetracarboxylic acid diimide (**5**) were synthesized according to the literature procedure^6^.^1^H NMR and ^13^C NMR spectra were recorded on a Bruker 300 MHz spectrometer with CDCl_3_ as the solvent. Fourier transform infrared (FT-IR) measurements were performed on a Bruker Tnsor-27 spectrophotometer. Mass spectra were recorded using a Bruker Maxis UHR-TOF mass spectrometer. Ultraviolet absorption spectra, and fluorescence measurements were recorded on a Varian CARY-50 spectrophotometer and a Hitachi FL-4500 spectrofluorometer, respectively.

### Fluorescence quantum yield measurements

The quantum yields of PDIs **1**, **2**, and **3** in solutions (10 μM in chloroform) were recorded on a Varian CARY-50 spectrophotometer and a Hitachi FL-4500 spectrofluorometer. The absorbance spectra were measured within a maximum absorbance close to 0.05 (*l* = 10 cm). All spectroscopic measurements were performed using a cuvette with a 1 cm path length at 25 °C. For each experiment, the slit width was 1.0 nm for both excitation and emission. Relative quantum efficiency was obtained according to the following equation

$${{\rm{\Phi }}}_{{\rm{sample}}}={{\rm{\Phi }}}_{{\rm{standard}}}({{\rm{A}}}_{{\rm{standard}}}{/{\rm{A}}}_{{\rm{sample}}})({{\rm{F}}}_{{\rm{sample}}}{/{\rm{F}}}_{{\rm{standard}}})({{{\rm{n}}}_{{\rm{sample}}}}^{2}{{/{\rm{n}}}_{{\rm{standard}}}}^{2})$$

where “Φ” is the quantum yield, “A” is the absorbance at the excited wavelength, “F” is the integrated area under the emission curve, and “n” is the refractive index of the solvent used (chloroform, 1.445; water, 1.333). Fluorescein (Φf = 0.85) in 0.1 M aqueous NaOH was used as fluorescence standard, and the excited wavelength is 508 nm, which is the intersection point of fluorescein and compound PDIs **1**–**3** in low concentration.

### Cyclic voltammetry (CV) measurements

Cyclic voltammograms (CV) were performed with a three electrode electrochemical cell on a CH 1604C electrochemical analyzer at a scan rate of 20 mV/s and the current sensitivity was given as 0.01 μA, polished 2 mm glassy carbon as working electrode, Ag/AgNO_3_ as reference electrode and Pt as counter electrode. Tetra (n-butyl) ammonium hexafluorophosphate (TBAPF_6_) solution (0.1 M) was used as the supporting electrolyte.

### Scanning electron microscopic measurements

SEM were performed with a FEI NOVA NANOSEM 450 microscope. The samples were prepared by casting one drop of the nanostructure suspension onto a glass coverslip. The dried samples were coated with gold prior to the SEM imaging. The fluorescence microscopic images and optical microscopic images were performed on an Olympus (Japan) BH2 fluorescence microscope, which provides excitation in the range of 450–490 nm. The quantum yields in the solid states were measured with the Hamamatsu spectrometer C11347 Quantaurus-QY. Fluorescence lifetimes in solutions (10 μM in DCM) were measured with the Hamamatsu spectrometer C11367.

### Computational details

The theoretical study for structure optimization and the property calculations were performed on the standard 6–31 G^*^(d) basis setusing the Becke’s three parameter gradient-corrected hybrid density function B3LYP method .All the calculations were performed using the Gaussian03 program installed on a Windows PC.

### Synthesis and characterization

The synthetic routes of PDIs **1**, **2**, and **3** (Fig. S1) along with their characterization data are reported in the Supplementary Methods.

### Synthesis of PDI 1

Compound **5** (120 mg, 0.2 mmol), phenol (95 mg, 1.0 mmol), 200 mg K_2_CO_3_ and the catalyzed KI were suspended in 10 mL anhydrous N-methylpyrrolidone (NMP). The resulting mixture was stirred at 25 °C for 6 h under argon atmosphere, and then poured into MeOH (7 mL) and 2 M HCl solution (30 mL). The precipitate was collected by vacuum filtration, washed with methanol, and then dried in vacuum. The crude product was further purified by silica gel column chromatography with eluent dichloromethane/petroleum ether (4:1 by volume). After solvent was removed, a red solid of 116 mg (90%) **1** was obtained^1^.H-NMR (300 MHz, CHCl_3_, TMS, ppm): *δ* = 9.44 (d, *J* = 6.0 Hz, 1 H), 8.62–8.51 (m, 5 H), 8.19 (s, 1 H), 7.45 (m, 2 H), 7.30 (m, 1 H), 7.15 (m, 2 H), 5.01 (m, 2 H), 2.53–2.36 (m, 4 H), 1.91–1.75 (m, 8 H), 1.43–1.25 (m, 8 H).FT-IR (KBr, cm^−1^): *v* = 2931, 2849, 1696, 1649, 1601, 1482, 1402, 1338, 1251, 1187, 813, 757. MS (MALDI-TOF): m/z 647.2 [M + H]^+^.

### Synthesis of PDI 2

The compound **2** was synthesized according to the procedure of synthesis of synthesis of **1**. Briefly, 120 mg (0.2 mmol) compound **5** and 130 mg (1.0 mmol) p-chlorophenol reacted at room temperature for 6 h in NMP to yield **2** in 92%^1^.H-NMR (300 MHz, CHCl_3_, TMS, ppm): *δ* = 9.28 (d, *J* = 6.0 Hz, 1 H), 8.70–8.31 (m, 5 H), 8.10 (s, 1 H), 7.43 (d, *J* = 9.0 Hz, 2 H), 7.09 (d, *J* = 6.0 Hz, 2 H), 5.01 (m, 2 H), 2.58–2.49 (m, 4 H), 1.93–1.62 (m, 8 H), 1.41–1.25(m, 8 H)0.^13^C NMR (75 MHz, CDCl_3_, ppm): *δ* = 163.79, 163.58, 163.56, 162.86, 155.42, 153.26, 133.99, 133.84, 133.07, 131.68, 130.73, 130.68, 130.52, 129.72, 128.82, 128.30, 128.23, 126.54, 125.60, 124.73, 124.05, 123.55, 123.40, 123.27, 123.11, 122.20, 120.93, 54.21, 54.04, 29.11, 29.09, 26.56, 26.51, 25.46, 25.43. FT-IR (KBr, cm^−1^): *v* = 2927, 2851, 1698, 1648, 1585, 1483, 1410, 1322, 1257, 1195, 807, 742, 648. MS (MALDI-TOF): m/z 681.2 [M + H]^+^.

### Synthesis of PDI 3

Compound **5** (0.2 g, 0.34 mmol) and K_2_CO_3_ (200 mg) were suspended in 12 mL chloroform and 8 mL n- propanol. The reaction mixture was refluxed for 10 h under argon atmosphere. After being cooled to room temperature, the solution was filtrated and evaporated to dryness. The crude product was purified by silica gel column chromatography with the eluent CH2Cl2/petroleum ether 4:1 to give a red solid **3** (90 mg, 45%)., Characterization data: ^1^H-NMR (CDCl_3_, 300 MHz, TMS, ppm): *δ* = 8.57 (m, 3 H), 8.46-8.35 (m, 3 H), 8.23 (s, 1 H), 4.96 (m, 2 H), 3.06 (m, 2 H), 2.50 (m, 5 H), 1.74 (m, 4 H), 1.64 (m, 6 H), 1.41 (m, 8 H), 0.80–1.00 (m, 2 H).^13^C NMR (75 MHz, CDCl_3_, ppm): *δ* = 163.64, 139.58, 130.47, 129.24, 128.41, 127.27, 126.51, 125.96, 123.11, 122.72, 121.99, 54.18, 37.95, 29.13, 26.60, 25.49, 21.91, 13.61. FT-IR (KBr, cm^−1^): *v* = 2922, 2845, 1691, 1651, 1611, 1415, 1343, 1307, 1257, 1179, 1000, 894, 853, 804, 735, 629, 578, 448, 416. MS (APCI): m/z 612.2 M^−^.

## Electronic supplementary material

Supplementary data
