# Supplementary material for: Self-assembly, optical and electrical properties of perylene diimide dyes bearing unsymmetrical substituents at bay position
Source: Sci Rep. 2018 May 29;8:8208. doi: 10.1038/s41598-018-26502-5 (PMC5974014; doi:10.1038/s41598-018-26502-5)

**Supporting Information**

Self-assembly, optical and electrical properties of perylene diimide dyes bearing unsymmetrical substituents at bay position

Fengxia Zhang ^1, 4^, Yongshan Ma ^2, 5^*, Yanhui Chi ^1^, Haohai Yu ^3^, Yanan Li ^1^, Tianyi Jiang ^2^, Xiaofeng Wei ^2^, and Jingmin Shi ^1^*

*^1^College of Chemistry, Chemical Engineering and Materials Science, Collaborative Innovation Center of Functionalized Probes for Chemical Imaging in Universities of Shandong, Key Laboratory of Molecular and Nano Probes, Ministry of Education Shandong Provincial Key Laboratory of Clean Production of Fine Chemistry, Shandong Normal University, jinan 250014, P. R. China. E-mail: shijingmin1955@163.com.*

*^2^ School of Municipal and Environmental Engineering, Shandong Jianzhu University, Jinan 250101，P. R. China. E-mail:* [*mlosh@sdjzu.edu.cn*](mailto:yanhuichi@126.com;).

*^3^ State Key Laboratory of Crystal Materials Shandong University Jinan 250100, P. R. China.*

*^4^ Shandong Provincial Key Laboratory of Metrology and Measurement, Shandong Institute of Metrology, Shandong Social Justice Institute of Metrology, Jinan 250014, P. R. China.*

*^5^ Co-Innovation Center of Green Building, Jinan 250101，P. R. China.*

*Correspondence and requests for materials should be addressed to Y. M. (email: mlosh@sdjzu.edu.cn)

**Experimental section**


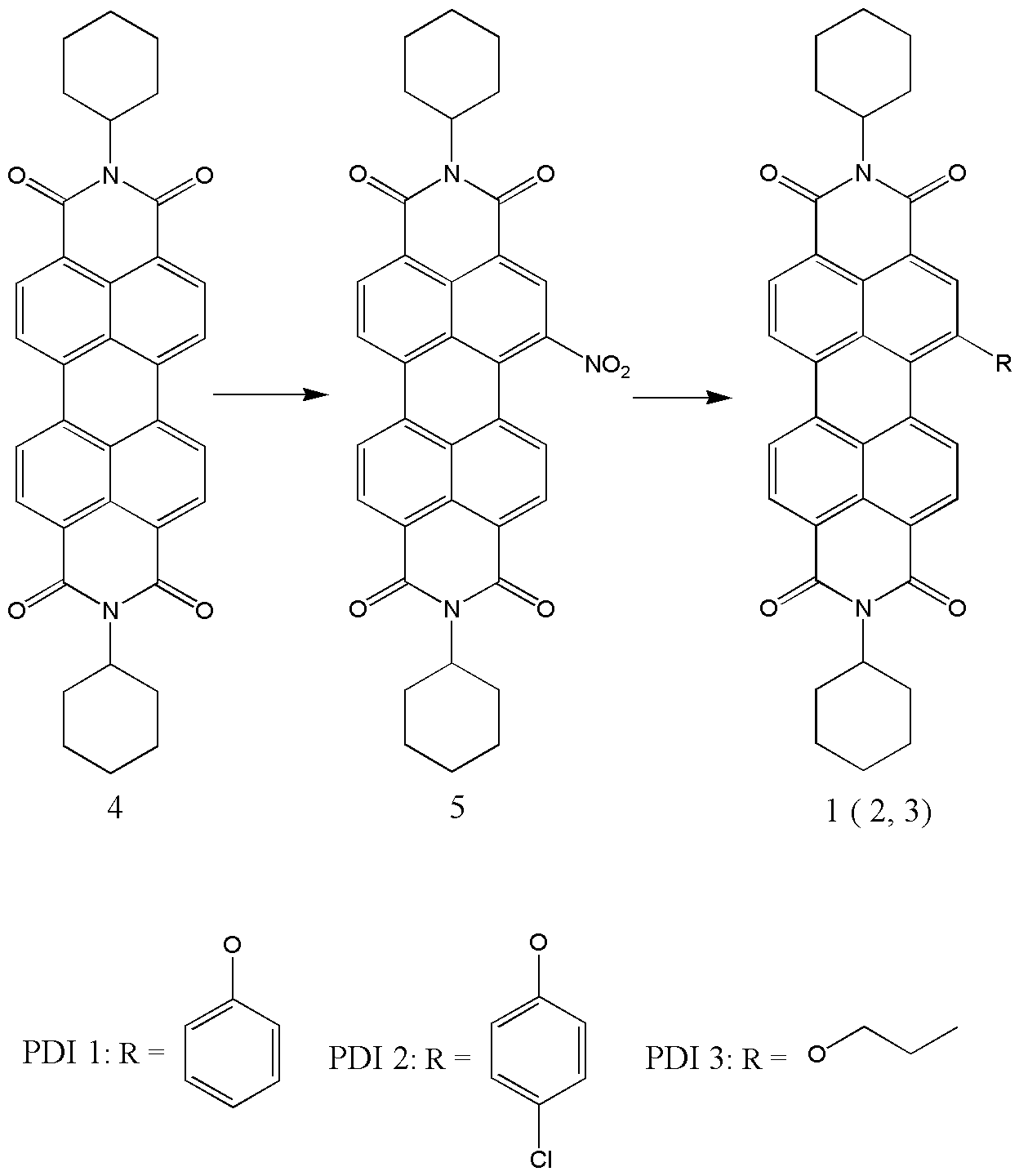


Fig.S-1 Synthesis routes of PDI 1-3.

**Spectra of NMR, FTIR and MALDI-TOF-MS**


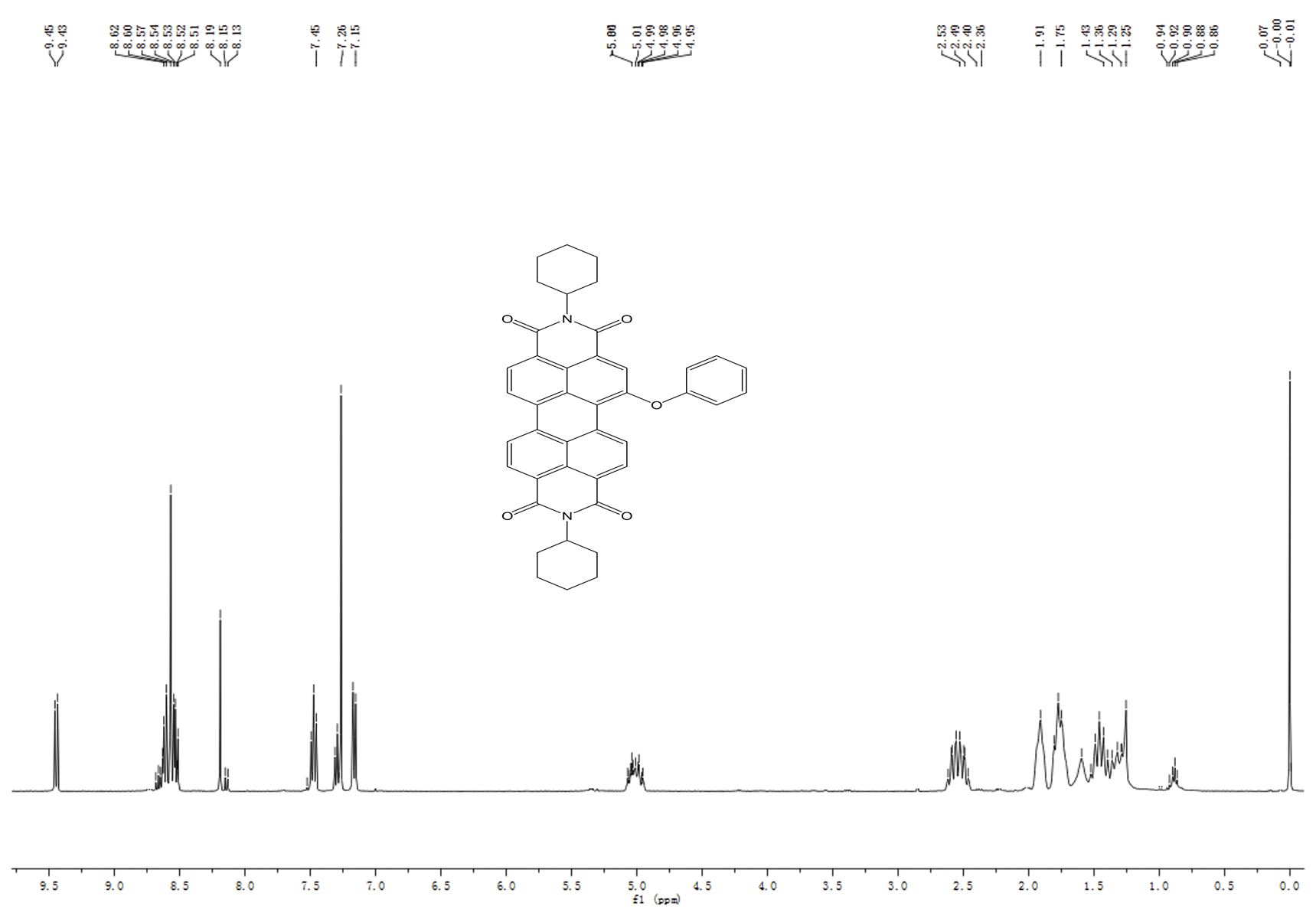


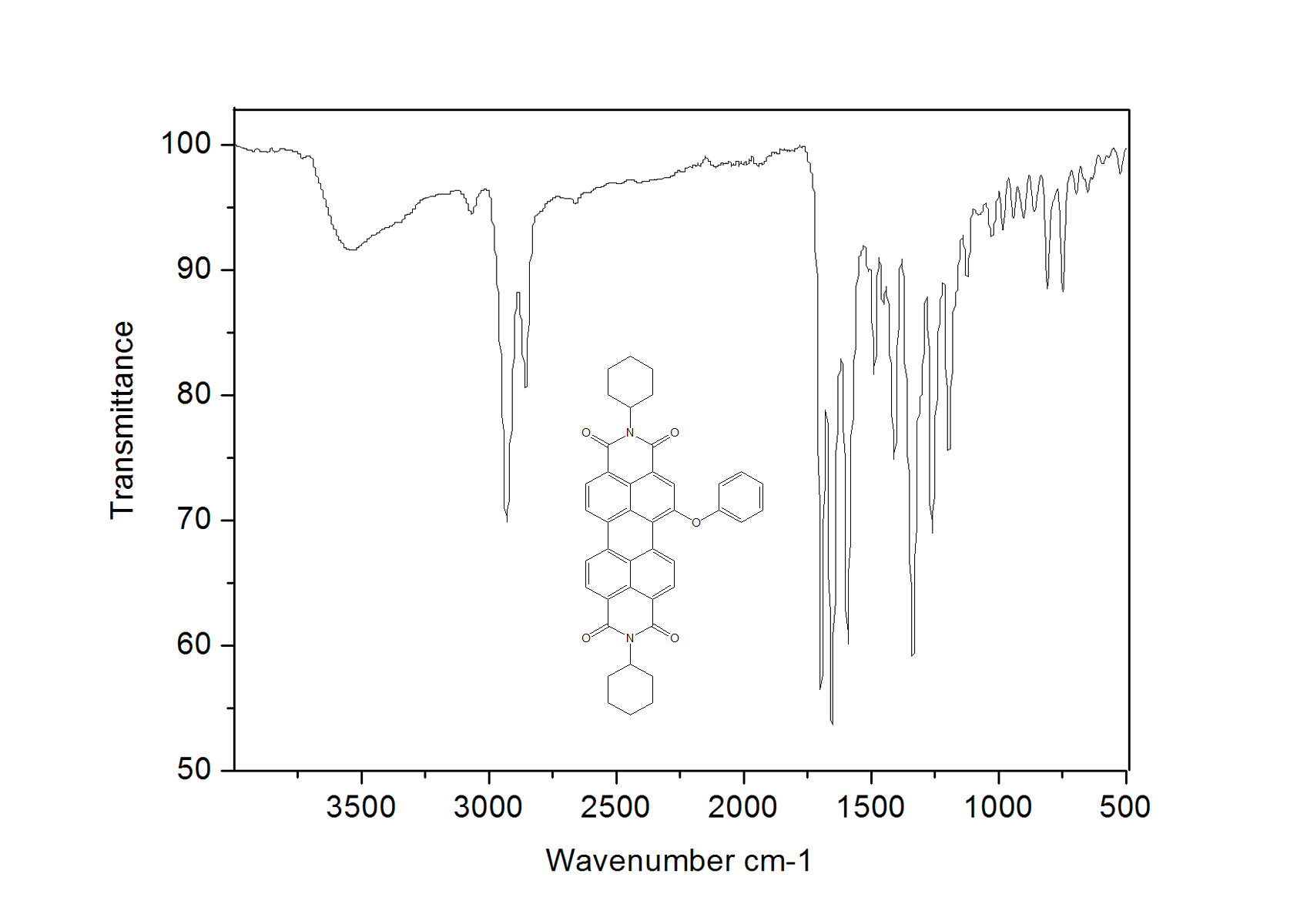


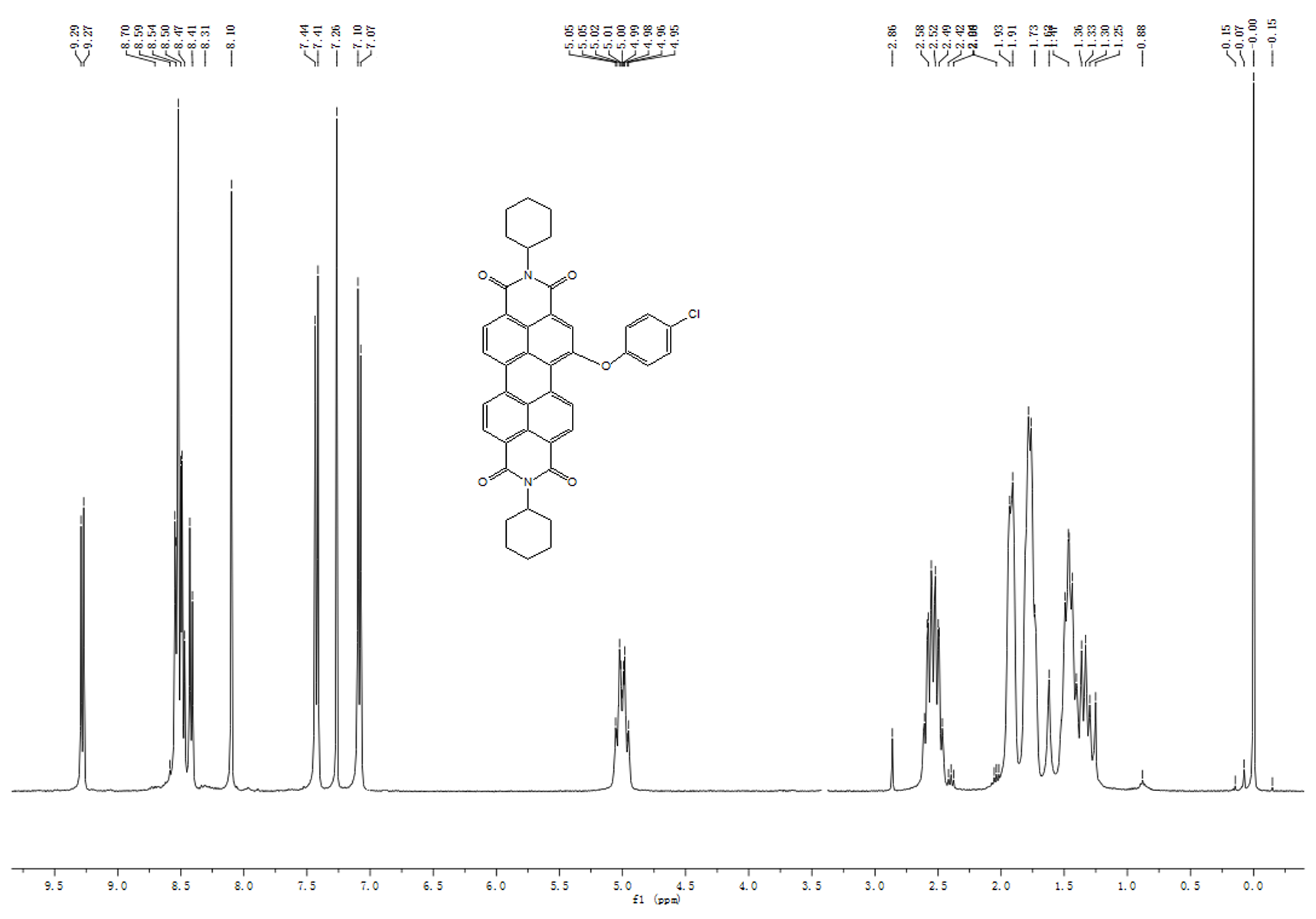


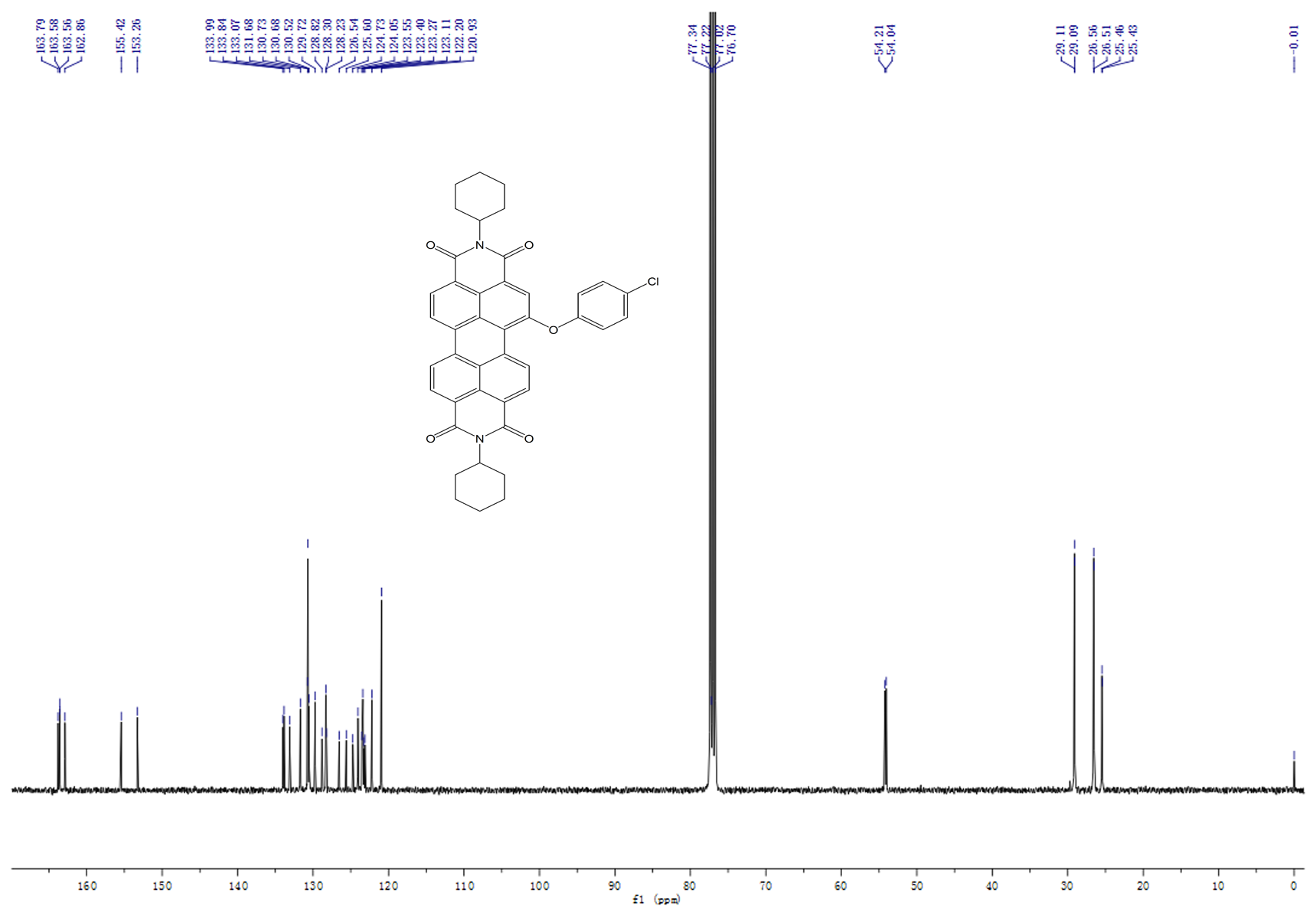


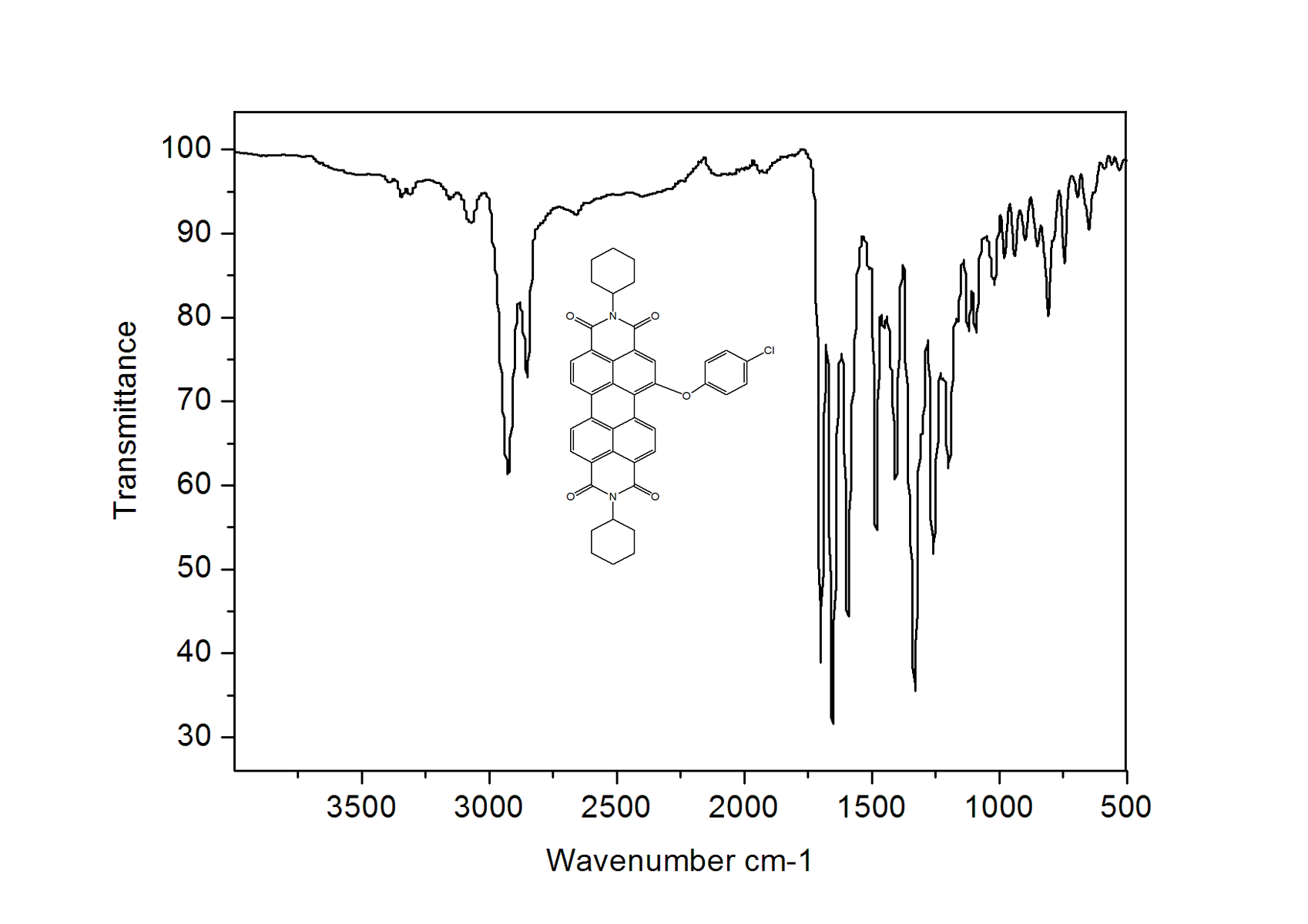


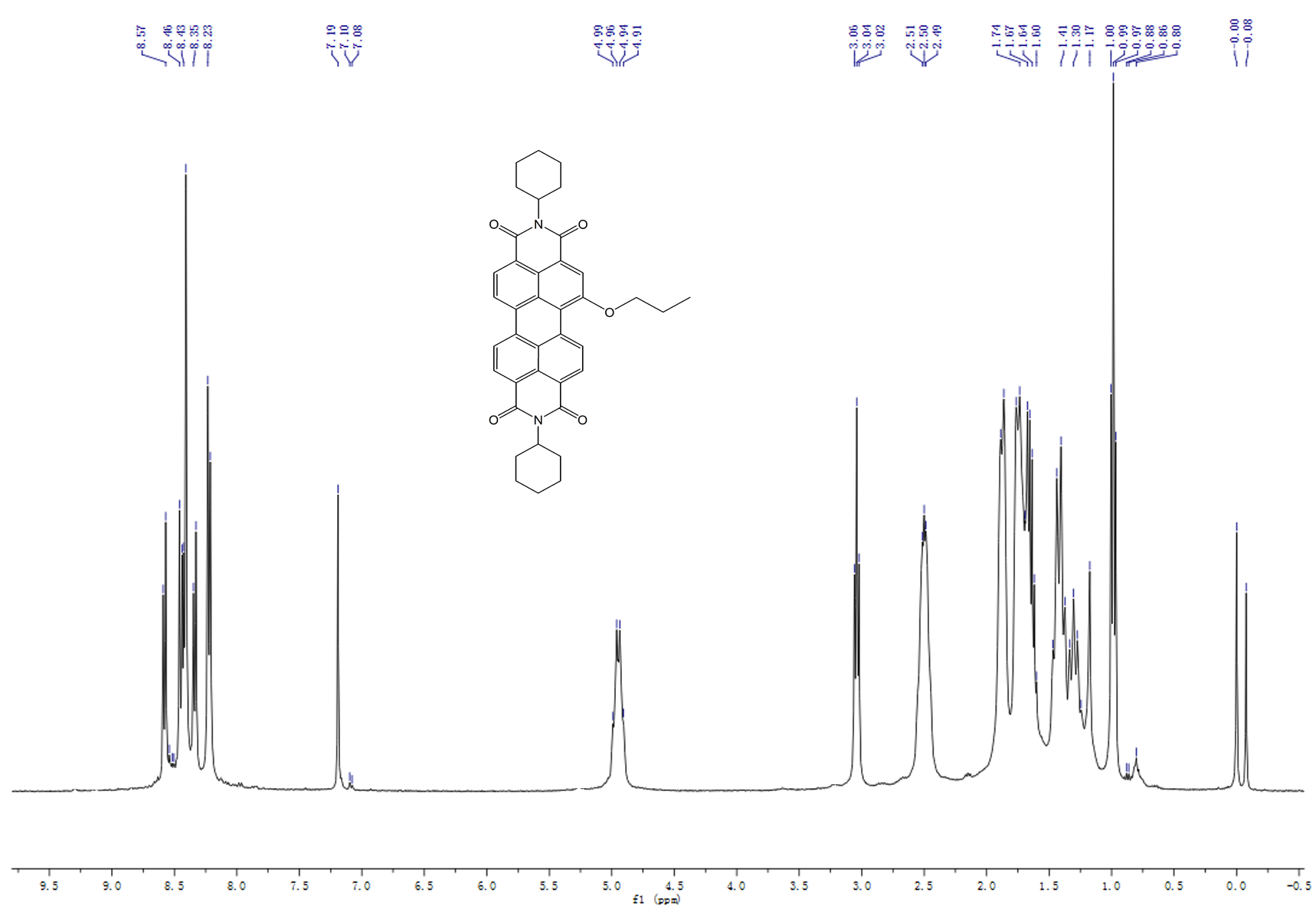


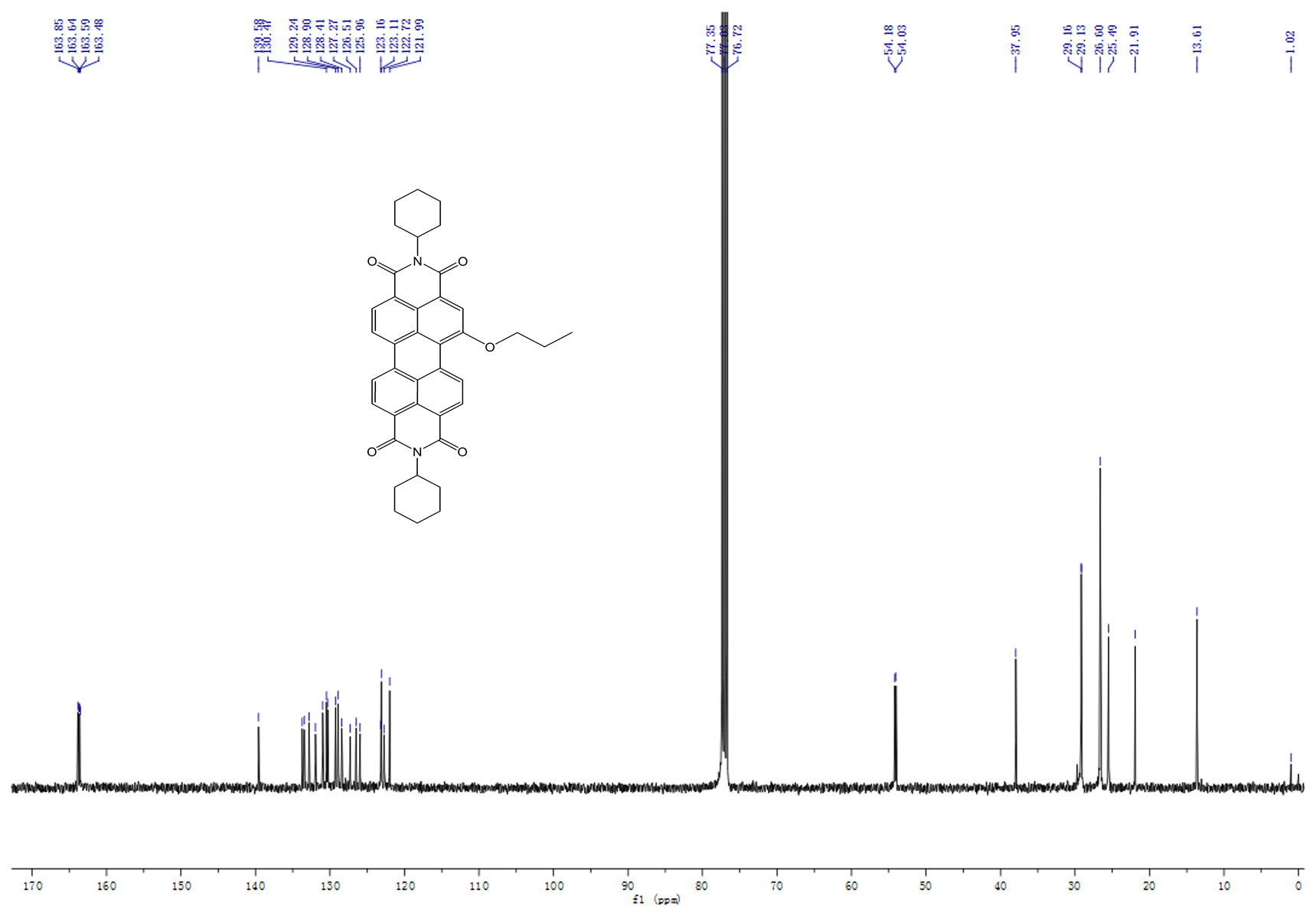


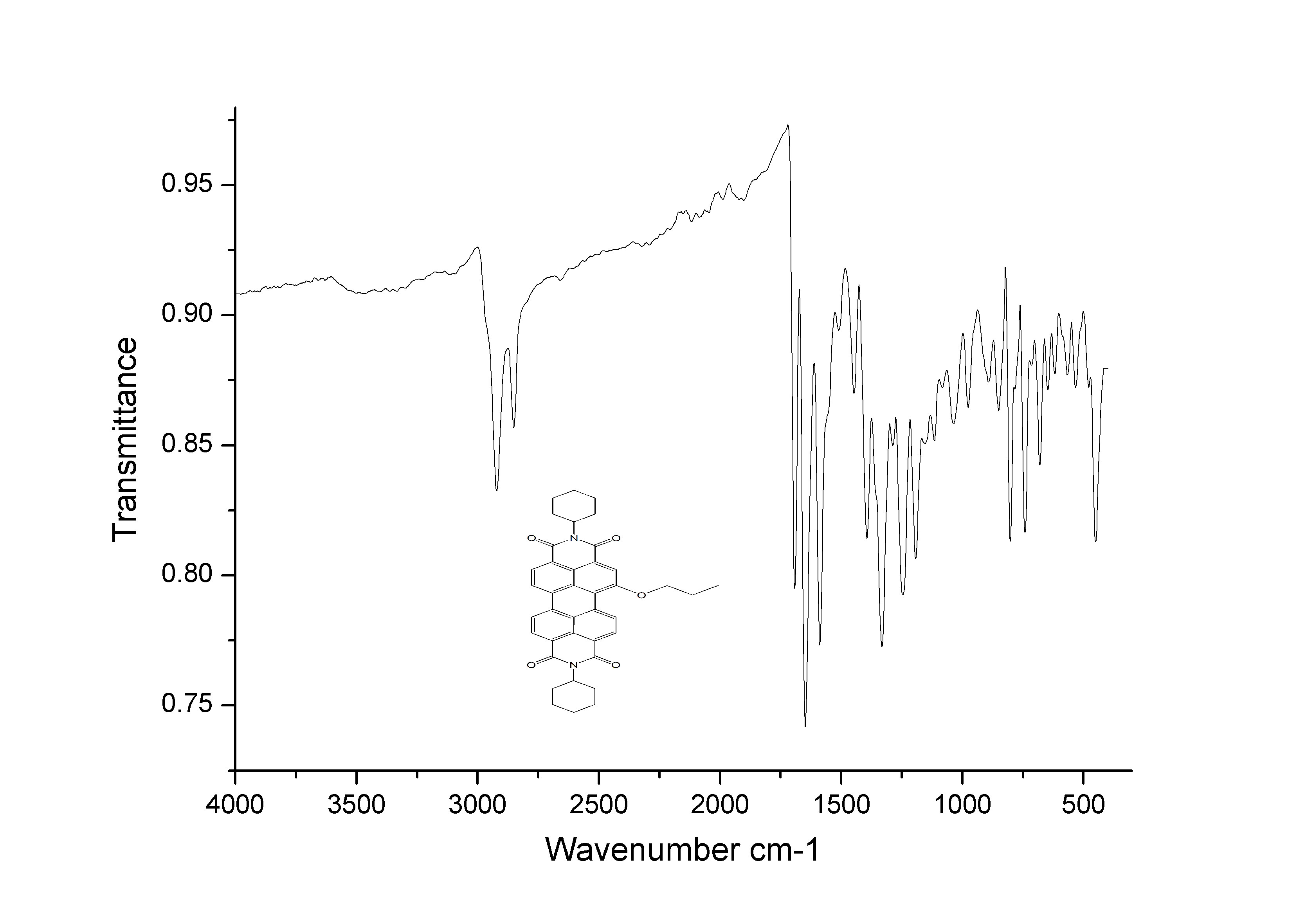


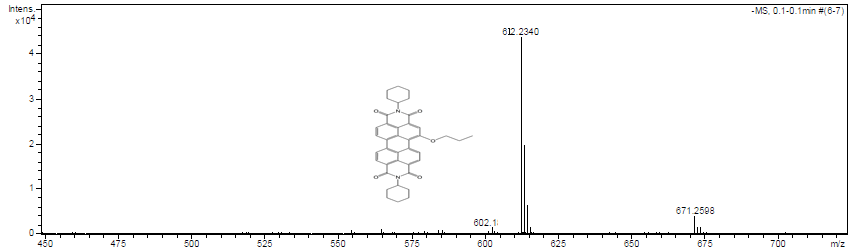


Fig. S-2 The fluorescence emission spectra of the PDIs **1**, **2** and **3** in solid state.


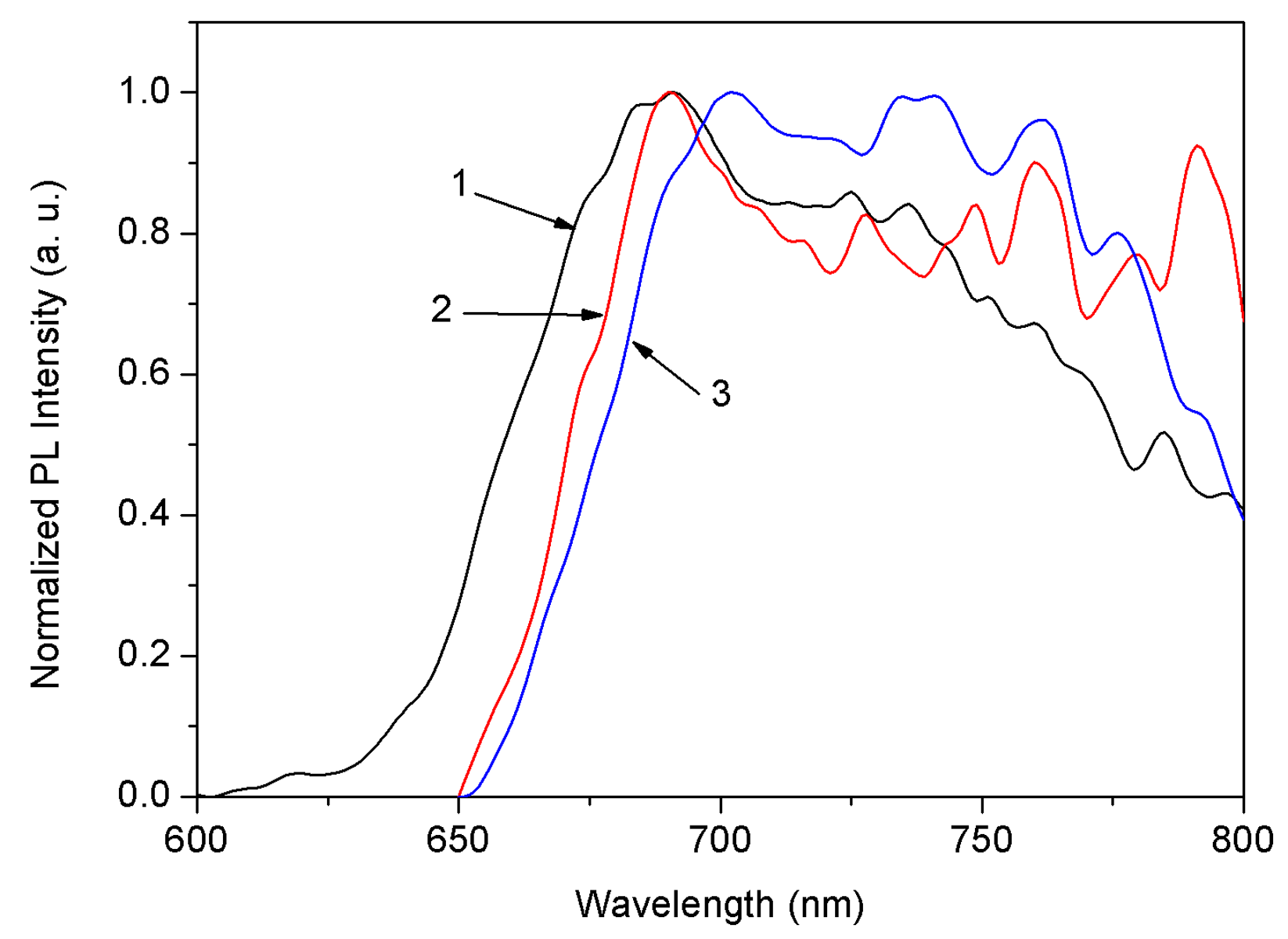


Fig. S-3 Normalized absorption spectra of 1 (a), 2 (b) and 3 (c) in dichloromethane at various concentrations.


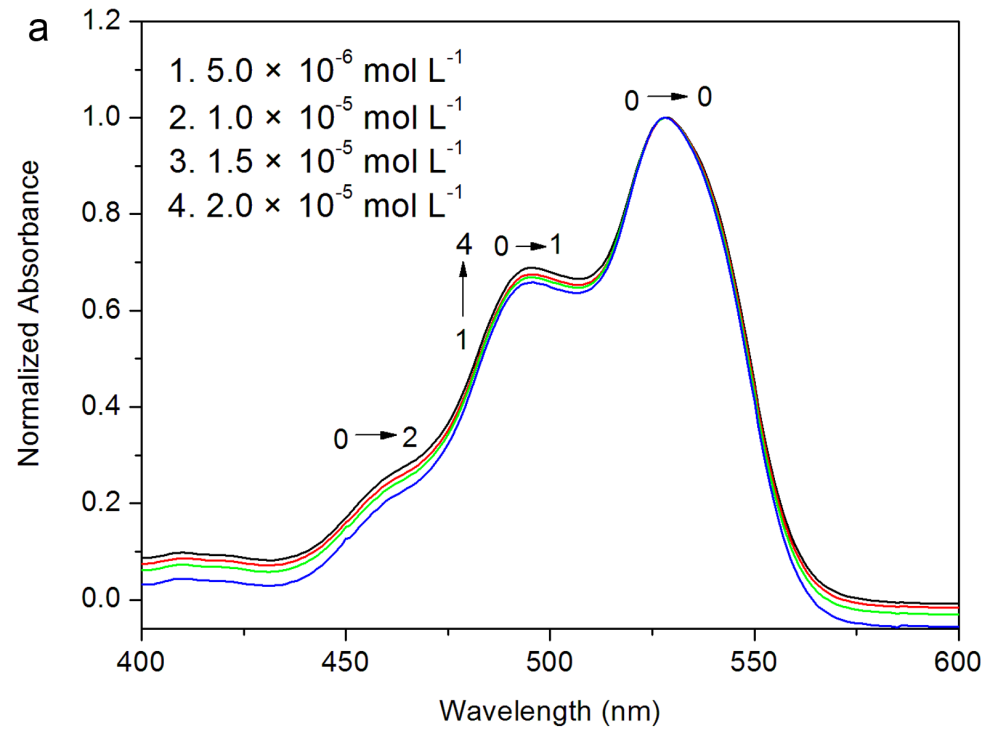


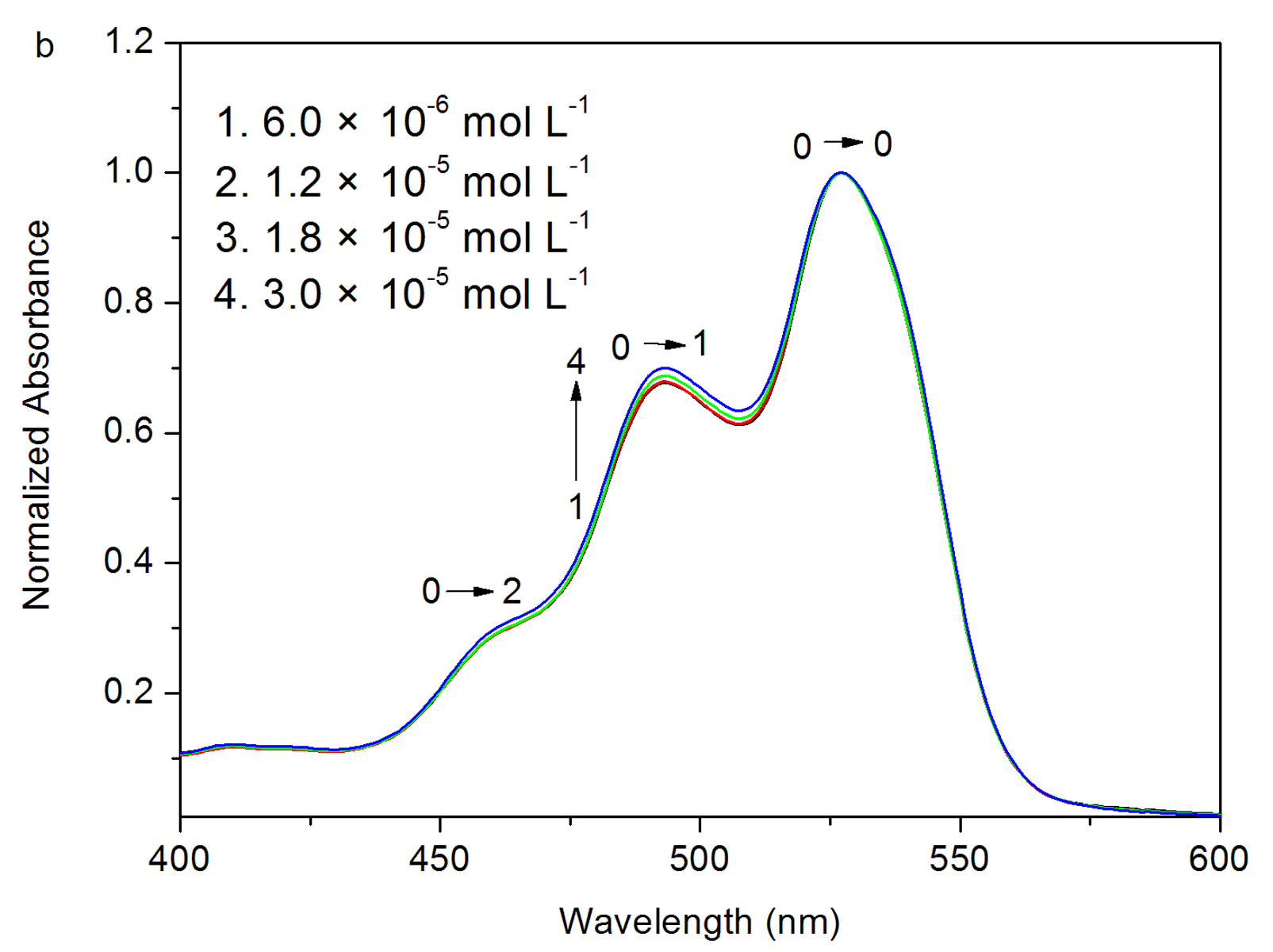


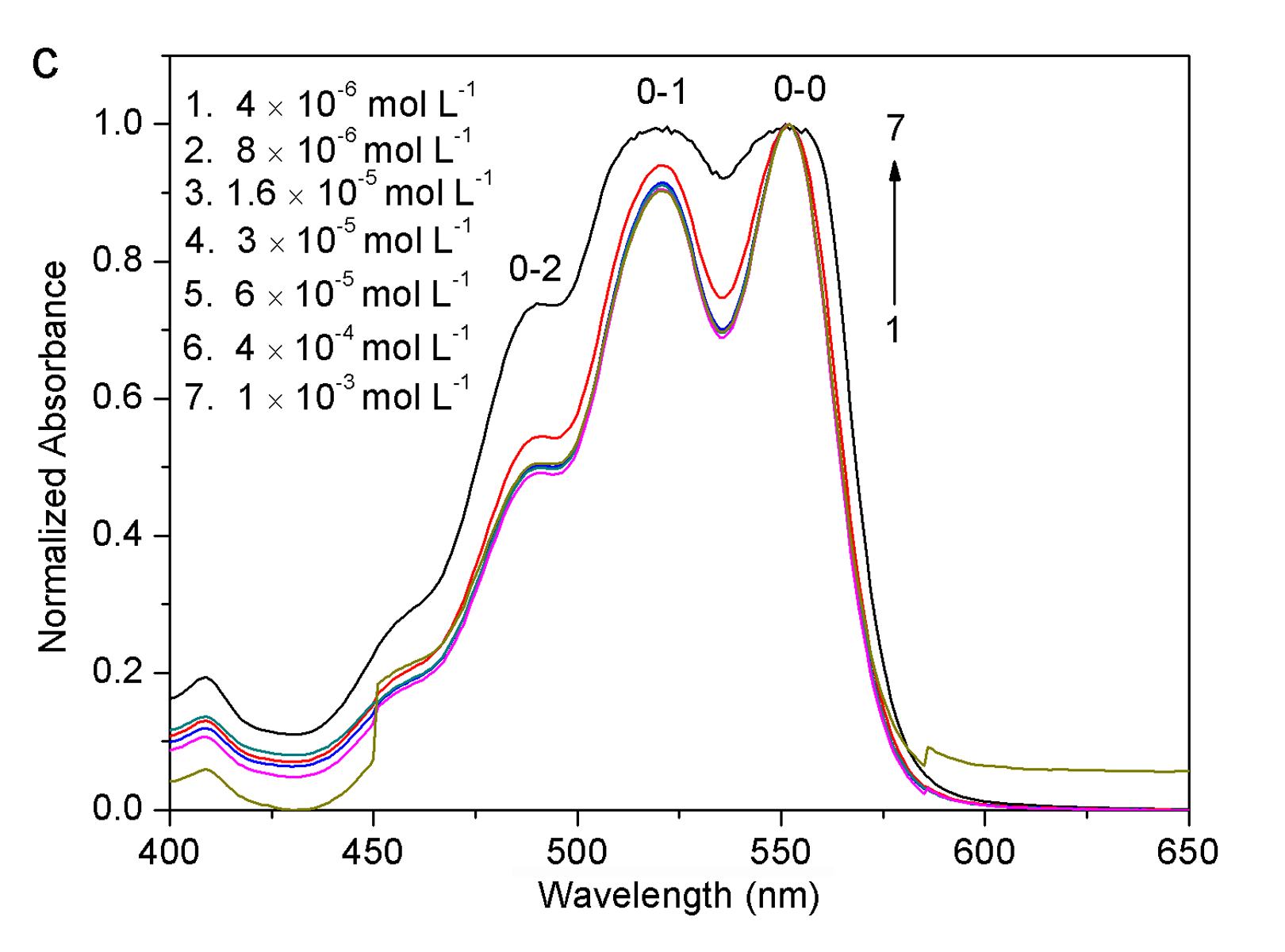


Fig. S-4 Lifetime decay curve of (a) PDI 1, (b) PDI 2 and (c) PDI 3.


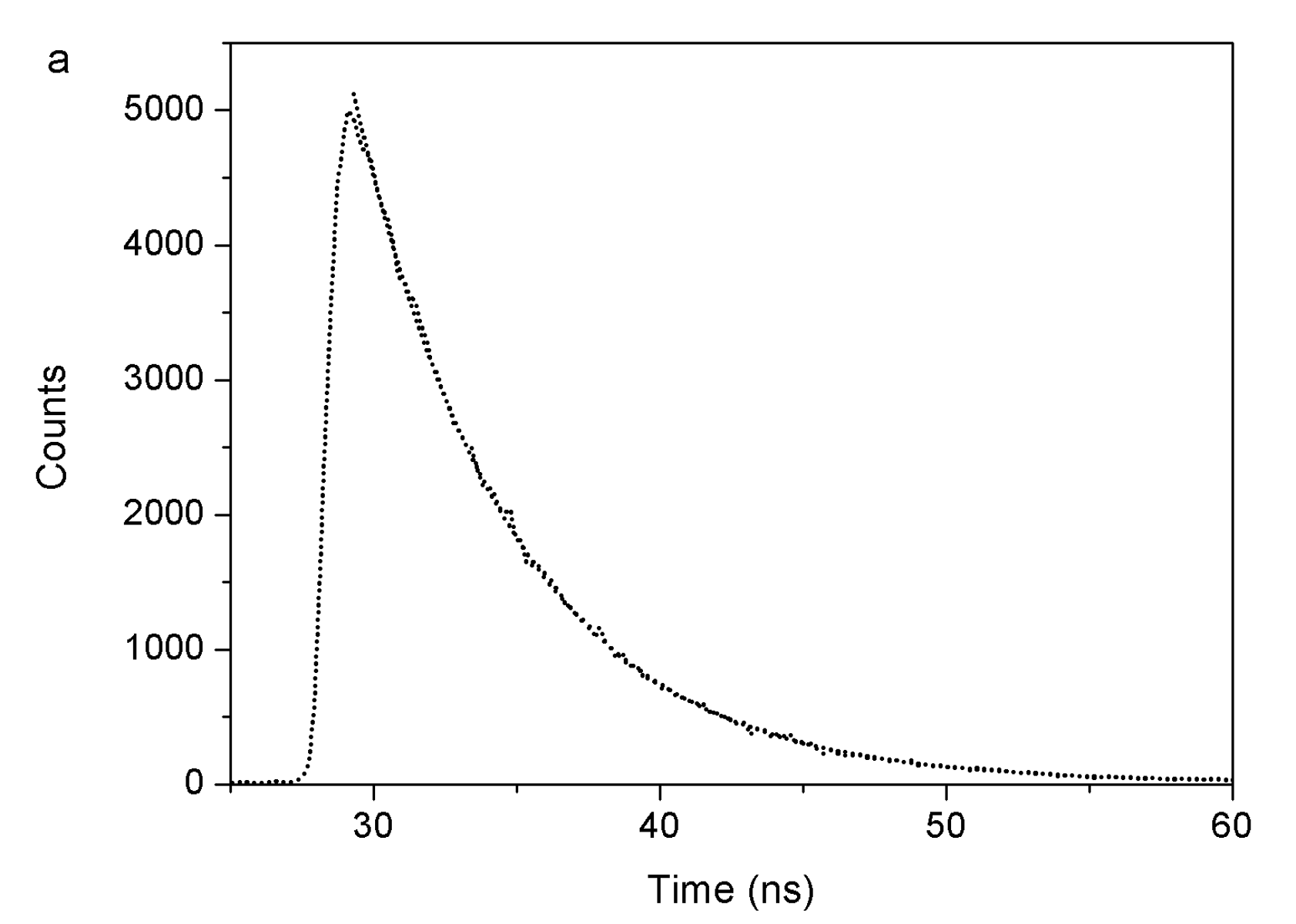


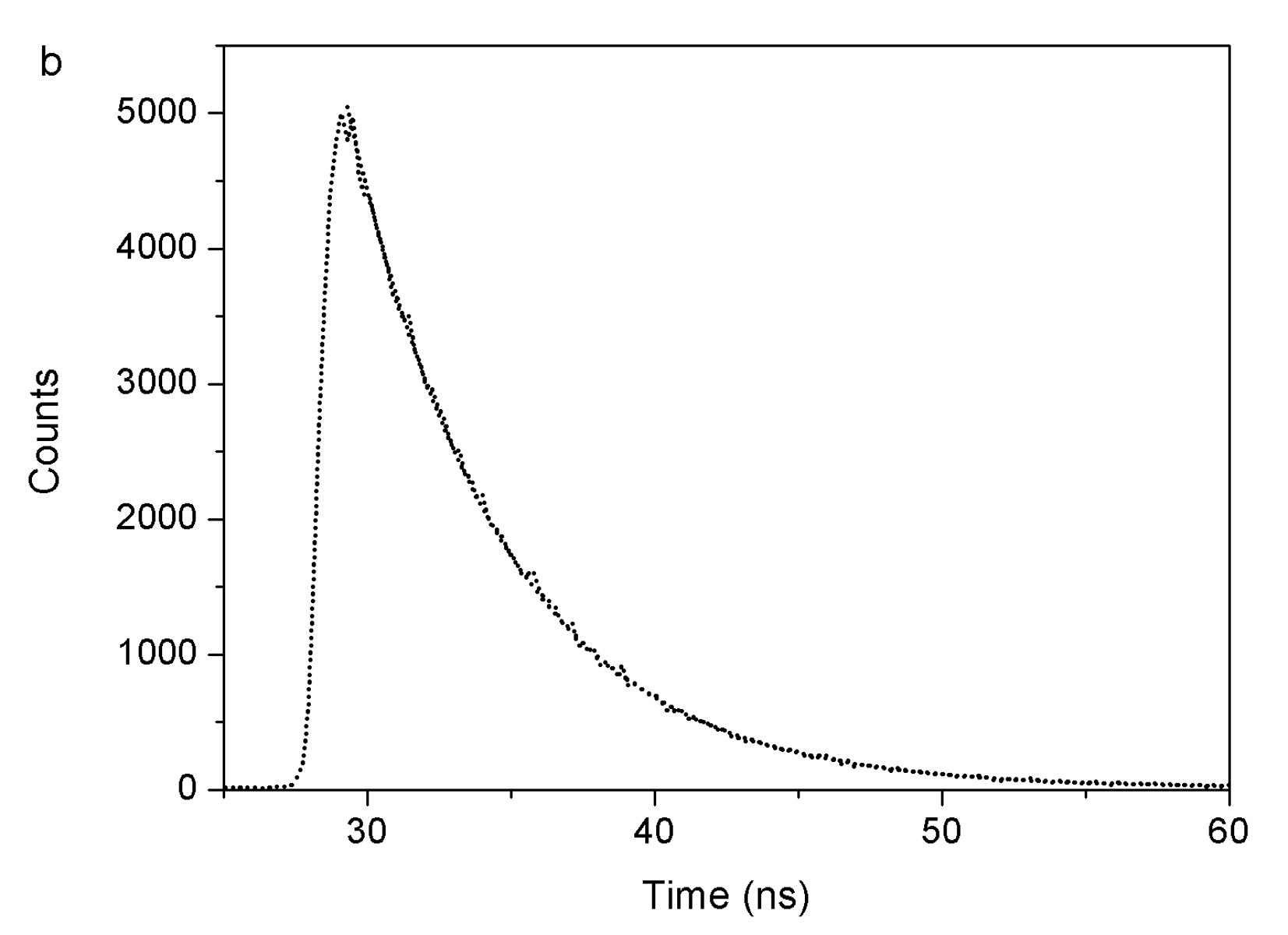


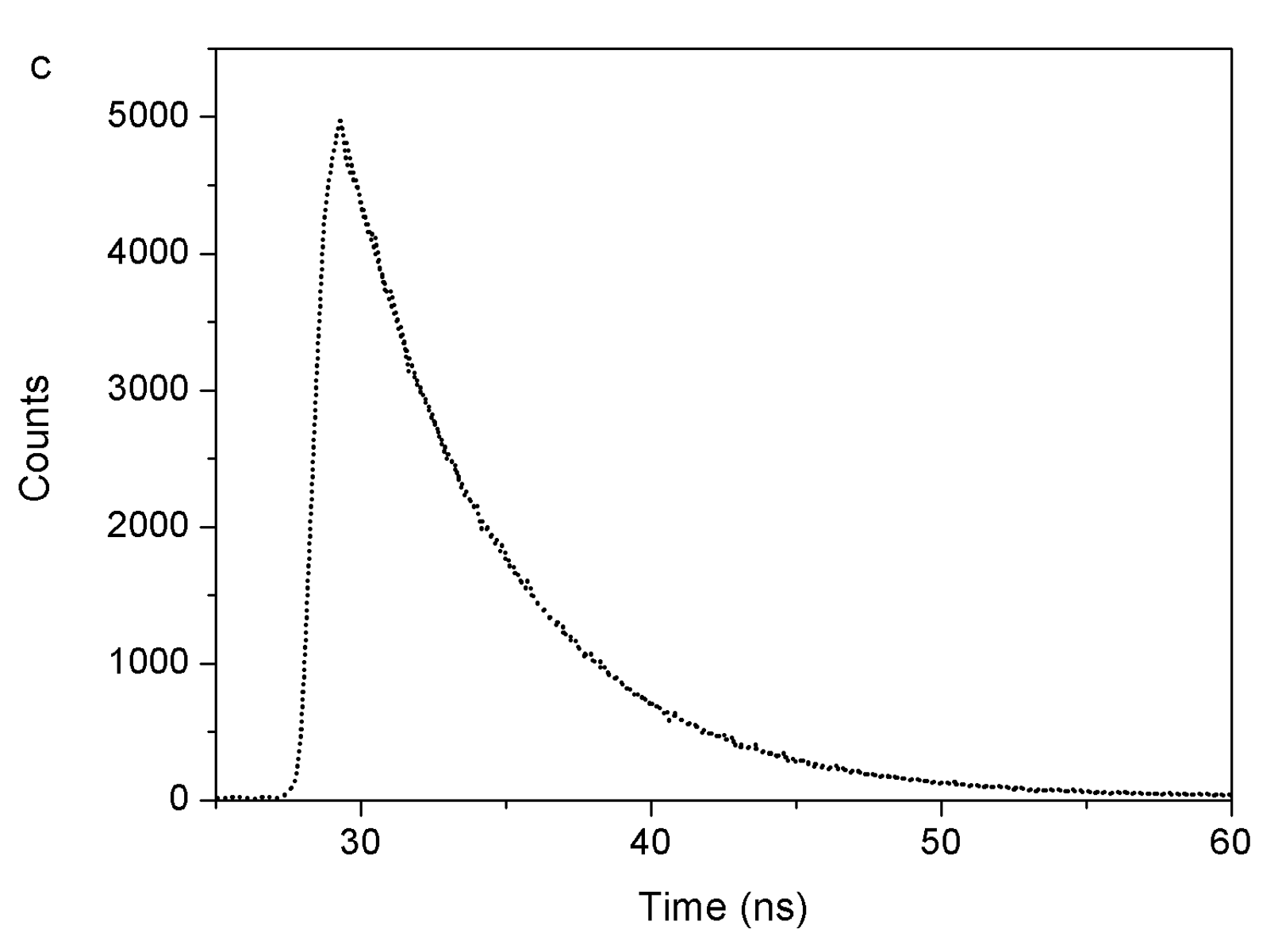

Supplement: Supplementary file 1 — Supplementary data [file 41598_2018_26502_MOESM1_ESM.doc]
